# Systems Analysis of Early Host Gene Expression Provides Clues for Transient *Mycobacterium avium* ssp *avium* vs. Persistent *Mycobacterium avium* ssp *paratuberculosis* Intestinal Infections

**DOI:** 10.1371/journal.pone.0161946

**Published:** 2016-09-21

**Authors:** Sangeeta Khare, Kenneth L. Drake, Sara D. Lawhon, Jairo E. S. Nunes, Josely F. Figueiredo, Carlos A. Rossetti, Tamara Gull, Robin E. Everts, Harris. A. Lewin, Leslie Garry Adams

**Affiliations:** 1 Department of Veterinary Pathobiology, College of Veterinary Medicine & Biomedical Sciences, Texas A&M University, College Station, Texas, 77843, United States of America; 2 Division of Microbiology, National Center for Toxicological Research, US Food and Drug Administration, Jefferson, Arkansas, 72079, United States of America; 3 Seralogix, LLC, Austin, Texas, 78730, United States of America; 4 Department of Animal Sciences, University of Illinois at Urbana-Champaign, Urbana, Illinois, 61801, United States of America; Rutgers Biomedical and Health Sciences, UNITED STATES

## Abstract

It has long been a quest in ruminants to understand how two very similar mycobacterial species, *Mycobacterium avium* ssp. p*aratuberculosis* (MAP) and *Mycobacterium avium* ssp. *avium* (MAA) lead to either a chronic persistent infection or a rapid-transient infection, respectively. Here, we hypothesized that when the host immune response is activated by MAP or MAA, the outcome of the infection depends on the early activation of signaling molecules and host temporal gene expression. To test our hypothesis, ligated jejuno-ileal loops including Peyer’s patches in neonatal calves were inoculated with PBS, MAP, or MAA. A temporal analysis of the host transcriptome profile was conducted at several times post-infection (0.5, 1, 2, 4, 8 and 12 hours). When comparing the transcriptional responses of calves infected with the MAA versus MAP, discordant patterns of mucosal expression were clearly evident, and the numbers of unique transcripts altered were moderately less for MAA-infected tissue than were mucosal tissues infected with the MAP. To interpret these complex data, changes in the gene expression were further analyzed by dynamic Bayesian analysis. Bayesian network modeling identified mechanistic genes, gene-to-gene relationships, pathways and Gene Ontologies (GO) biological processes that are involved in specific cell activation during infection. MAP and MAA had significant different pathway perturbation at 0.5 and 12 hours post inoculation. Inverse processes were observed between MAP and MAA response for epithelial cell proliferation, negative regulation of chemotaxis, cell-cell adhesion mediated by integrin and regulation of cytokine-mediated signaling. MAP inoculated tissue had significantly lower expression of phagocytosis receptors such as mannose receptor and complement receptors. This study reveals that perturbation of genes and cellular pathways during MAP infection resulted in host evasion by mucosal membrane barrier weakening to access entry in the ileum, inhibition of Ca signaling associated with decreased phagosome-lysosome fusion as well as phagocytosis inhibition, bias toward Th2 cell immune response accompanied by cell recruitment, cell proliferation and cell differentiation; leading to persistent infection. Contrarily, MAA infection was related to cellular responses associated with activation of molecular pathways that release chemicals and cytokines involved with containment of infection and a strong bias toward Th1 immune response, resulting in a transient infection.

## Introduction

*Mycobacterium avium* ssp *paratuberculosis* (MAP) infection in cattle is established following the ingestion of bacteria, invasion of the intestinal mucosa, and subsequent events of the host-pathogen interaction. These bacteria invade M cells and macrophages, and are capable of resisting host defenses, and multiply to reach very high intracellular numbers leading to chronic granulomatous enteritis known as “Johne’s disease” in cattle. *Mycobacterium avium* ssp *avium* (MAA) is antigenically and genetically similar organism to MAP, but is relatively nonpathogenic for cattle [1–3]. MAP infection may have severe economic impact in dairy, as well as, meat production industry. This economic loss may be due to the low milk and meat production, weight loss, premature culling, increased mortality, infertility, predisposition to other diseases, and cost associated with replacement of animals, diagnostic tests administration, veterinary care, animal welfare and public-health issues [4, 5]. The phylogenetic analysis of *Mycobacterium avium* subspecies revealed that MAP and MAA do not cluster together, rather they are independently evolved clones [6, 7]. Upon interaction with the immune cells, MAP may be killed or survive in the macrophages. If MAP survives in the macrophage, it triggers adaptive immune responses and persists in the infected animals. This persistent infection typically mounts an effective systemic immune response; however, this occurs during the late stage of infection and results in enteric granulomatous inflammation. Whereas MAA infection to cattle generally results in an effective immune response and elimination of pathogen. Despite 95% genomic similarity between MAP and MAA, what causes the persistent vs. transient infection in cattle is not fully understood. Several studies have focused on the role of mononuclear phagocytes during MAP and MAA infection [8–11]. These studies showed that MAA was a stronger activating factor for phagocytic cells than MAP. Furthermore, MAP causes only an early transient activation of MAPK-p38 in bovine mononuclear phagocytes that may be a key mechanism involved in the capacity of MAP to survive in bovine monocytes. Bovine monocytes or monocyte-derived macrophages incubated with MAP lead to a rapid phosphorylation of MAPK-p38, higher expression of IL-10, and failure to acidify phagosomes or kill the organisms [12]. In contrast, MAA-infected monocytes expressed low levels of IL-10, partially acidified phagosomes, and killed approximately half of the organisms within 96 hours. The outcome of the disease depends on the interaction and collective response of various cell types that are present at the mucosal surface. However, little emphasis has been given to the other cell types during MAP or MAA infection. Moreover, which components of host response are involved in the activation of innate immunity is not very well defined in chronic infections. Thus, a more comprehensive knowledge is needed regarding the pathogen interaction within the enteric milieu that includes several other cell types, such as, enterocytes, M cells, dendritic cells, goblet cells, endothelial cells, natural killer cells, mast cells and more. Toward this goal, we utilized the calf ligated jejuno-ileal loop model for analyzing early changes at the mucosal surface during MAP infection [13, 14]. Aim of the present study was to compare temporal gene expression during MAP and MAA infection in the calf jejuno-ileal mucosa, including Peyer’s patches of calves infected with MAP or MAA. This comparison was done against same control. Thus, this analysis provided us specific gene that were differentially regulated during infection with MAP or MAA; so we were able to generate a comparative analysis regarding persistent vs transient responses. A systems biology approach was applied to analyze the data which enabled us to predict specific cell type involvement as well as specific cellular pathway perturbations during the early infection. Furthermore, we identified innate immune signatures (mechanistic genes) sufficient to envisage the subsequent adaptive immune response leading to persistent or transient infection by MAP and MAA respectively.

## Materials and Methods

Most of the materials and methods are essentially the same as described earlier by our group [14]. For clarity of this publication some portions are reproduced from the earlier article and cited accordingly.

### Culture of MAP and MAA

*Mycobacterium avium* ssp *paratuberculosis* (ATCC 19698 from American Type Culture Collection, Manassas, VA), was grown at 37°C in 7H9 broth (Difco Laboratories, Detroit, MI) supplemented with 2.5% (vol/vol) glycerol (Sigma Chemical Co., St. Louis, MO), oleic acid-albumin-dextrose-catalase (Difco Laboratories, Detroit, MI), 0.05% Tween 80 (Sigma Chemical Co., MO), and 2 mg of Mycobactin J (Allied Monitor, Inc., Fayette, MO). *Mycobacterium avium* ssp *avium* (ATCC 35716 from American Type Culture Collection, Manassas, VA) was grown at 37°C in 7H9 broth supplemented with 2.5% (vol/vol) glycerol, oleic acid-albumin-dextrose-catalase and 0.05% Tween 80. For inoculation of MAP and MAA, single-cell suspensions and counting was done as described previouly [15].

### Animals

Three to four weeks old, clinically healthy male Holstein-Friesian calves (n = 4) weighing 45–55 kg, were used in the experiment under an approved animal use protocol in accordance with animal use policy under the supervision of the Texas A & M University Institutional Animal Care and Research Advisory Committee (AUP 2007–70). The calves were fed antibiotic-free milk replacer twice daily and water *ad libitum*. Animals included in this study were culture negative for the presence of *Salmonella* spp. and pre-existence of mycobacterial species [14, 15].

### Bovine Ligated Jejuno-ileal Loop Surgery

The method used for the bovine ligated jejuno-ileal loop surgery was almost the same as described previously [14]. Briefly, the neonatal calves were fasted for 24 hours prior to the non-survival surgery, anesthetized and maintained analgesic for the course of the 12 hr experiment [14]. The abdominal wall was clipped, prepared aseptically with chlorhexidine and isopropanol prior to opening. The abdominal wall was incised and the entire length of the Peyer’s patch of the distal jejunum and ileum exteriorized. Eighteen 6–8 cm loops of the distal jejunum and ileum were ligated with umbilical tape with 1–2 cm interloop spaces continuously. Individual loops were injected with 1.0 ml of PBS containing 3x10^9^ CFU of MAP or MAA. The same volume of sterile PBS was inoculated into the control loops. The loops were replaced into the abdominal cavity, and the incision temporarily closed with Backhaus towel clamps. Intravenous 2.5% dextrose and 0.45% normal physiological was used to maintain circulating blood volume at 5 ml/kg/hour. At 0.5, 1, 2, 4, 8 and 12 hours after bacterial inoculation, one each of control, MAP and MAA loops were excised. Samples for bacteriologic culture and RNA extraction were collected as described below. Electrocautery was used to control hemorrhage after excision of loops. During the entire length of experimental procedure, the calves were monitored for vital signs (anesthesia depth, temperature blood pressure, heart rate and hydration status,). At the end of the experiment (12 hours post infection), the calves were humanely euthanized with a single bolus dose (60mg/lb IV) of pentobarbital sodium.

### Bacteriology

Two mucosal tissue punches (6 mm biopsy punch) were collected from each loop (for bacteriology. Mucosal tissue punches (two tissue punches from each loop) were washed three times in PBS, weighed, homogenized in PBS, and serially diluted. The tissue extracts were plated in triplicate onto Herrold Egg Yolk Media containing ANV (Becton Dickinson and Company, Sparks, MD) with or without Mycobactin J (for the culture of MAP or MAA respectively) and incubated at 37°C. The cultures were observed visually weekly for any contamination, and the final counts of colony forming units were recorded on week 16. Colonization of tissue was considered to be positive when bacterial colonies were detected. Tissue burden was defined as the number of colony forming units per milligram of tissue. The statistical significance of differences was calculated using two-tailed Student’s *t* test.

### Extraction and Quality Analysis of RNA

RNA was extracted from the mucosal biopsy punches collected at 30 minutes and at 1, 2, 4, 8, and 12 hr post-infection. In brief, tissue was immediately minced and transferred to Trizol reagent (Molecular Research Center, Cincinnati, OH). Tissues were further disrupted with hand-held mechanical tissue grinder equipped with a RNase, DNase free plastic disposable pestle. The RNA extraction was performed using the recommended protocol from the manufacturer (Molecular Research Center, Cincinnati, OH). The resultant RNA pellet was re-suspended in DEPC-treated water (Ambion, Austin, TX). Genomic DNA was removed by RNase-free DNase I treatment (DNA-free, Ambion) according to the manufacturer’s instructions, and samples were stored at −80°C until used. RNA concentration was quantified by measuring absorbance at λ260nm using a NanoDrop^®^ ND-1000 (NanoDrop, Wilmington, DW). RNA quality was determined using a Nano-Chip^®^ on an Agilent 2100 Bioanalyzer (Agilent, Palo Alto, CA). All the RNA samples used in this study were of good to excellent quality (results not shown). Samples were characterized by distinct 18S and 28S rRNA peaks and RNA size distribution. Ten micrograms of RNA were used as starting material for each microarray. Bovine reference RNA was used as a control for each microarray [14]. Individual microarrays for individual calves and individual time point were performed (4 animals * 6 time points * 3 test material = 72 microarrays)

### Microarray Sample Preparation and Hybridization to Bovine cDNA Microarray

Labeling of cDNA and hybridization to microarray has been described, previously [14, 16, 17]. Briefly, the total RNA was reverse transcribed using Superscript III reverse transcriptase (Invitrogen, Carlsbad, CA) and labeled with amino-allyl-UTP (Ambion, Austin, TX). Cy3 and Cy5 dye esters were covalently linked to the amino-allyl group by incubating the samples with the dye esters in 0.1M sodium carbonate buffer. cDNA from bovine experimental samples (i.e. from MAP or MAA infected) and PBS control loops were co-hybridized against cDNA generated from the bovine reference RNA sample 13K bovine 70mer oligoarray. Prior to hybridization, the microarrays were denatured by exposing to steam from boiling water for three seconds, UV cross-linked and then immersed in pre-hybridization buffer [5X sodium chloride, sodium citrate buffer (SSC), 0.1% sodium dodecyl sulfate (SDS) (Thermo Fisher Scientific, Waltham, MA), 1% bovine serum albumin (BSA)] at 42°C for a minimum of 45 min followed by four washes in RNase-, DNase-free, distilled water, immersion in 100% isopropanol for 10 seconds, and dried by centrifugation. Slides were hybridized at 42°C for approximately 40 hr in a dark humid chamber (Corning, Corning, NY) and washed for 10 min at 42°C with low stringency buffer [1 X SSC, 0.2% SDS] followed by two 5 min washes in a higher stringency buffer [0.1 X SSC, 0.2% SDS and 0.1 X SSC] at room temperature in dark with mild agitation.

### Data Acquisition

Immediately after washing, the slides were scanned using a GenePix 4100 laser scanner (Axon Instruments Inc., Foster City, CA). The spots representing genes on the arrays were adjusted for background and normalized to internal controls using image analysis software (GenePixPro 4.0; Axon Instruments Inc.). Spots with fluorescent signal values below background were disregarded in all analyses.

### Microarray Data Analysis

The analysis of microarray data utilized a suite of systems biology computational tools integrated within the Systems Biology Computational Pipeline (SBCP) (Seralogix LLC, Austin, Texas). SBCP facilitates an approach employing Bayesian methods called Dynamic Bayesian Gene Group Activation (DBGGA) for pathway and Gene Ontology (GO) analysis and modeling at the systems biology level offering an integrated view of biological mechanisms and networks of interactions. DBGGA determines pathway/GO activations and repressions as well as mechanistic genes, which are genes that contribute the most to the perturbation of a pathway in context within the network and gene-to-gene relationships. The SBCP integrates data from several different heterogeneous database sources such as Gene Ontology (GO) Consortium [18], Genbank (www.ncbi.nlm.nih.gov/Genbank), Ensembl, KEGG [19–22], and others such that the cumulative modeling information provides greater biological insight. For differential gene analysis, the SBCP employs a Bayesian estimation method with variance smoothing to compute Z-scores (Bayesian Z-score), fold change and p-values. Details of these methods have been described earlier [14, 23] and a brief overview provided in S1 File.

### Microarray data deposited in the Gene Expression Omnibus

All microarray data were deposited in the Gene Expression Omnibus at the National Center for Biotechnology Information (www.ncbi.nlm.nih.gov/geo/) Accession **#** GSE13888.

## Results

### Invasion of Jejunal and Jejuno-ileal Mucosa by MAP and MAA

**Fig 1:**
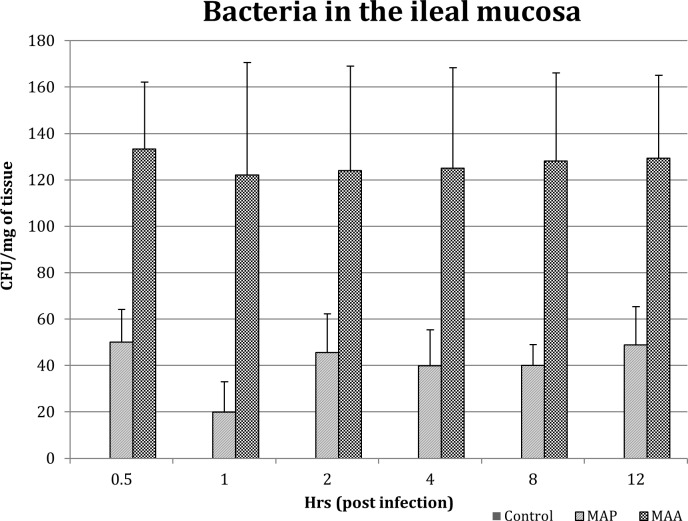
Invasion of Jejunal and Jejuno-ileal Mucosa by MAP and MAA.

MAP and MAA were recovered from the MAP-inoculated jejuno-ileal tissues at all the time points post infection (Fig 1). No MAP or MAA were detected in the PBS inoculated loops. No statistically significant changes in the number of MAP or MAA were detected in infected jejunal and jejuno-ileal tissues at any times post-inoculation (0.5–12 hours), however, MAA recovery was higher as compared to MAP from all the tissue samples.

### Sequential changes in the transcriptional profile of bovine host Peyer’s patches inoculated with MAP or MAA

Global gene expression analysis was performed using the RNA extracted from the mucosa of the Peyer’s patch of the distal small intestine (jejunum and ileum) of four calves at six time points post inoculation (30 minutes, 1, 2, 4, 8 and 12 hours) with MAP or MAA and compared with gene expression in loops inoculated with PBS (for same time point) as an experimental control. For the normalization of microarray data, bovine reference RNA, as described earlier was used with each sample [14].

We analyzed the data to determine differences between experimental conditions (MAP vs PBS or MAA vs PBS) and obtain detailed insight into the temporal relationships of various genes using the SBCP Bayesian Z score technique (Table 1). These Z-scores were computed after outlier removal (≥ 3 std. dev.) and filtered based on a significance of |Z-score| ≥ 1.96 and a false discovery rate, FDR ≤ 0.05. The Bayesian Z score analysis demonstrated that the total number of unique differentially expressed genes changed during the MAP infection was nearly the same as that in MAA, 744 compared to 617 respectively (Table 1). Between these two groups of significant genes (inclusive of all time points), there were 106 common up regulated genes and 35 down regulated between MAP and MAA inoculated intestinal tissue. There were a fair number of genes both up regulated and down regulated in common at each time point (Table 1). Over the whole time-course, there were several genes that were unique to either MAP (622), or to MAA (484). The results suggest that there are significant temporal pattern and gene expression differences between the MAP and MAA groups which may underlie the persistent and transient natures of host infection, respectively. S1 and S2 Tables provide the complete list of Bayesian Z-scored genes with associated q-values over the whole time-course for MAP and MAA respectively.

**Table 1:** Analysis of number of expressed genes changed during the MAP and MAA infection.

Condition	Total Changed	Total Up/Down	0.5 hr	1 hr	2 hrs	4 hrs	8 hrs	12 hrs
MAP vs. PBS	744	560	141	37	70	20	258	66
		184	16	71	30	16	54	4
MAA vs. PBS	617	400	54	15	62	4	129	150
		217	27	33	16	6	82	62
Common in MAP & MAA	141	106	17	11	11	1	27	16
		35	4	13	5	2	9	1
Unique in MAP	622	468	124	26	59	19	231	50
		154	12	58	25	14	45	3
Unique in MAA	484	304	37	4	51	3	102	134
		180	23	20	11	4	73	61

Bayesian Z-score (filtered at |zscore| ≥ 1.96) with variance smoothing, outlier removal, and false discovery rate ≤ 0.5 shows the analysis of number of expressed genes changed during the MAP and MAA infection. Note: |z-score| > = 1.96, maximum false discovery rate at any one time point Q-value < = 0.05 (or < = 5%)

### Distinct Differences between MAP and MAA Infected Host Pathway and GO Category Perturbations Support the Hypothesis of Persistence vs. Transient Mechanisms

It is assumed that the pathways and GO differences in activation and repression over the time course of infection provide evidence of plausible MAP/MAA host evasion and defensive mechanisms. Pathway and GO perturbation analysis was employed using SBCP DBGGA techniques to characterize the host response to MAP and MAA infection. As part of the DBGGA scoring method, the pathways, GO categories, and associated genes are scored based on the complete gene group and individual gene contribution to the pathway/GO perturbation. The DBGGA score is computed from the log-likelihood measure of the pathogen-infected condition data fit to the learned network model of the control (uninfected) condition as described in the S1 File. The log likelihood is transformed to a normalized Z-score, hereafter referred to as the pathway, GO, or individual gene DBGGA Z-score. The summary of scoring results (Table 2) of the pathways and GO categories indicated that both MAP and MAA groups had early pathway perturbations at 0.5 hours post infection. A comprehensive list of the significantly perturbed activated or repressed pathways, by time point, identified for MAP and MAA inoculated loops, with pathway DBGGA |Z-score| ≥ 2.24 threshold is provided in S3 Table and S4 Table, respectively. The GO DBGGA Z-score results for MAP and MAA are provided in S7 Table and S8 Table, respectively. These results are employed to identify contrasting differences and similarities as presented in the next section.

**Table 2:** DBGGA Scoring Summaries for Pathways and Gene Ontologies for both the MAP and MAA Conditions.

**Table 2(A) MAP Pathway**							
	**Time Post Innocation**						
**MAP Pathway DBGGA Scoring**	0.5 hr	1 hr	2 hrs	4 hrs	8 hrs	12 hrs	Total (Unique)
DBGGA Zscore Significantly Activated	47	0	2	3	13	126	144
DBGGA Zscore Significantly Repressed	41	0	1	0	0	18	59
Total Significantly Perturbed	88	0	3	3	13	144	162
**Table 2(B) MAA Pathway**							
	**Time Post Innocation**						
**MAA Pathway DBGGA Scoring**	0.5 hr	1 hr	2 hrs	4 hrs	8 hrs	12 hrs	Total (Unique)
DBGGA Zscore Significantly Activated	13	1	2	14	0	79	89
DBGGA Zscore Significantly Repressed	28	0	0	2	0	30	57
Total Significantly Perturbed	41	1	2	16	0	109	128
**Table 2(C) MAP GO Category Scoring Summary**							
	**Time Post Innocation**						
**MAP GO Category DBGGA Scoring**	0.5 hr	1 hr	2 hrs	4 hrs	8 hrs	12 hrs	Total (Unique)
DBGGA Zscore Significantly Activated	682	8	81	400	766	2152	2718
DBGGA Zscore Significantly Repressed	415	2	1	15	62	384	823
Total Significantly Perturbed	1097	10	82	415	828	2536	3138
**Table 2(D) MAA GO Category Scoring Summary**							
	**Time Post Innocation**						
**MAA GO Category DBGGA Scoring**	0.5 hr	1 hr	2 hrs	4 hrs	8 hrs	12 hrs	Total (Unique)
DBGGA Zscore Significantly Activated	194	62	375	1538	99	1451	2543
DBGGA Zscore Significantly Repressed	219	24	16	179	13	622	984
Total Significantly Perturbed	413	86	391	1717	112	2073	3001
**Table 2(E) MAP Pathway Gene DBGGA Scoring Summary**							
	**Time Post Innocation**						
**MAA Genes DBGGA Scoring**	0.5 hr	1 hr	2 hrs	4 hrs	8 hrs	12 hrs	Total (Unique)
DBGGA Zscore Significantly Up regulated	231	37	70	98	161	328	725
DBGGA Zscore Significantly Down regulated	253	25	48	45	64	228	591
Total Significantly Perturbed	484	62	118	143	225	556	1171
**Table 2(F) MAA Pathway Gene DBGGA Scoring Summary**							
** **	**Time Post Innocation**						
**MAA Genes DBGGA Scoring**	0.5 hr	1 hr	2 hrs	4 hrs	8 hrs	12 hrs	Total (Unique)
DBGGA Zscore Significantly Up regulated	141	64	134	193	69	270	688
DBGGA Zscore Significantly Down regulated	246	67	54	80	39	238	631
Total Significantly Perturbed	387	131	188	273	108	508	1144
**Table 2(G) MAP GO Gene DBGGA Scoring Summary**							
	**Time Post Innocation**						
**MAP GO Gene DBGGA Scoring**	0.5 hr	1 hr	2 hrs	4 hrs	8 hrs	12 hrs	Total (Unique)
DBGGA Zscore Significant Up Regulated	486	60	105	155	368	765	1531
DBGGA Zscore Significant Down Regulated	463	25	31	44	67	453	1038
Total Significantly Perturbed	949	85	136	199	435	1218	2381
**Table 2(H) MAA GO Gene DBGGA Scoring Summary**							
	**Time Post Innocation**						
**MAA GO Gene DBGGA Scoring**	0.5 hr	1 hr	2 hrs	4 hrs	8 hrs	12 hrs	Total (Unique)
DBGGA Zscore Significant Up Regulated	275	119	205	388	85	606	1422
DBGGA Zscore Significant Down Regulated	517	71	55	115	30	517	1220
Total Significantly Perturbed	792	190	260	503	115	1123	2381

Summary of significantly perturbed pathways, Gene Ontologies, and associated genes passing 97.5% confidence threshold (DBGGA |zscore| ≥ 2.24). This table compares: (A) MAP versus PBS (uninfected) pathway scores; (B) MAA versus PBS pathway scores; (C) MAP versus PBS Gene Ontology scores; (D) MAA versus PBS Gene Ontology scores; (E) MAP versus PBS Pathway gene scores; (F) MAA versus PBS Pathway gene scores; (G) MAP versus PBS Gene Ontology gene scores; and (H) MAA versus PBS Gene Ontology gene scores.

### Contrasting the Differences between MAP vs. MAA Mediated Host Responses

**Fig 2:**
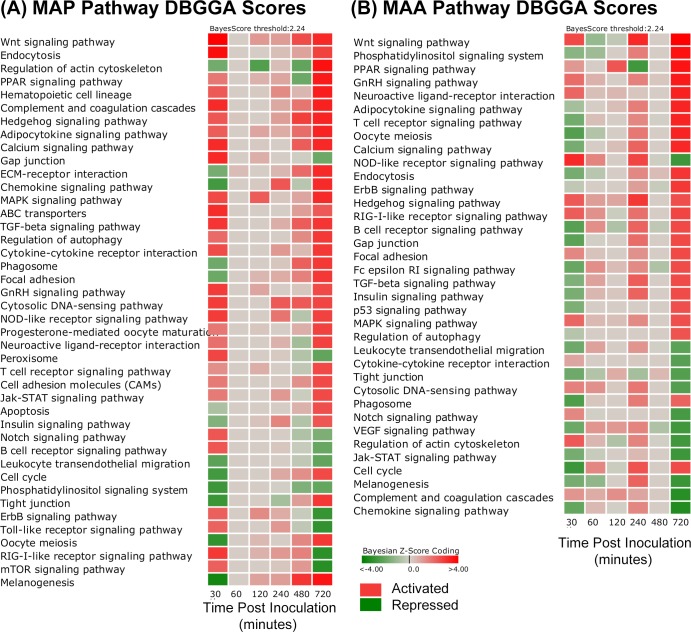
Heatmap comparison of significantly perturbed pathways (DBGGA |zscore| ≥ 2.24) for the (A) MAP vs PBS and (B) MAA vs PBS. Red indicates activation and green indicates repression. The gradient in color indicates magnitude of perturbation (i.e., higher |zscore|).

**Fig 3:**
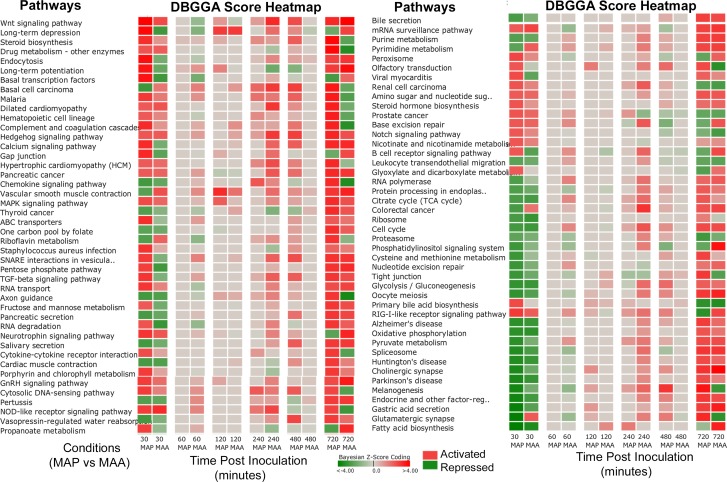
Heatmap of pathway scores of a side-by-side comparison (by time point) between MAP vs PBS versus MAA vs PBS. The heatmap compares MAP significantly perturbed pathways to the MAA condition. The selection of pathways was based on those that were found significant (DBGGA zscore ≥ 2.24) for the MAP condition at 30 minutes post infection. Red indicates activation and green indicates repression. The gradient in color indicates magnitude of perturbation (i.e., higher |zscore|).

**Fig 4:**
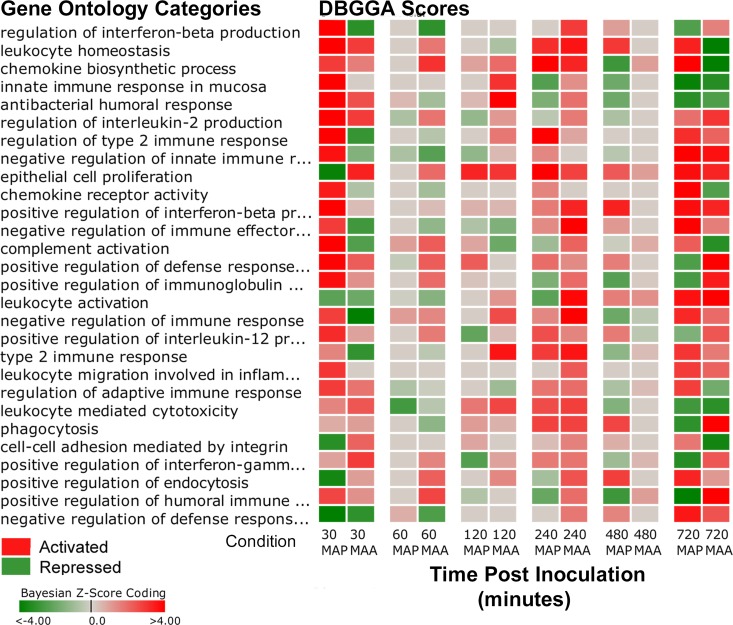
Heatmap of Gene Ontology scores of a side-by-side comparison (by time point) between MAP vs PBS versus MAA vs PBS. The heatmap compares MAP significantly perturbed Gene Ontology categories to the MAA condition. This subset of GO categories were selected based on relevance to the immune response and are top scoring GO categories found significant (DBGGA zscore ≥ 2.24) for the MAP condition at 30 minutes post infection. Red indicates activation and green indicates repression. The gradient in color indicates magnitude of perturbation (i.e., higher |zscore|).

The perturbed pathways and GO categories for MAP and MAA inoculated loops were rather different on a time-point to time-point basis and there were several distinct and interesting differences in the magnitude and state of their activation or repression. These differences can be observed in the pathway comparative heatmaps of Fig 2 in which the magnitude between MAP vs PBS (Fig 2A and MAA vs PBS (Fig 2B) can be observed for each time point in which red indicates pathway activation, green indicates repression, while grey indicates no significant difference. Fig 2A and 2B excluded metabolic pathways and diseases from the heatmap so that the results can be more focused on those associated with signaling cascades and cell and membrane interactions. It is apparent from Fig 2 and the summaries of Table 2 that both MAP and MAA had significant pathway perturbations at 0.5 and 12 hours post inoculation. However, in several cases, the pathway activations for MAP are opposite to that of MAA and in several cases the pathways are highly activated in one condition and not in the other. Fig 3 heatmap is a side-by-side comparison (by time point) that was based on first selecting all significantly perturbed MAP infected host pathways at 30 minutes post infection and then compared to MAA results. Likewise for the GO categories (top scoring and immune related) shown in Fig 4, there are striking differences in biological processes between MAP and MAA host response.

We focused our analysis to determine if these differences may lead to biologically significant persistence or transient outcomes during MAP/MAA infection. We compared the responses of the host against MAP and MAA by two different approaches. First, we performed a differential comparison of host responses based on the pathway and GO DBGGA analysis during MAP and MAA infection. Second, we identified the common and unique mechanistic genes (genes which contribute to perturbations in context with its interaction with other network genes or gene sets) for these pathways/GO categories during MAP and MAA infection.

#### Pathway and GO DBGGA Analyses

The temporal analysis of pathways and GO categories perturbed by MAP and MAA disclosed several similarities and some very distinct differences in the host responses. Pathways and GO categories that have either: 1) a reversed state of activation between MAP and MAA host response; or 2) have unique pathway/GO perturbations in comparison to the other infectious condition are hypothesized to be related to the MAP and MAA pathogenic differences. We employ the pathway DBGGA score heatmaps in Fig 3 to provide a quick visual comparison of significantly perturbed pathways for the MAP vs PBS and MAA vs PBS condition that focus on the early pathway activations of MAP at 30 minutes post infection. In general, MAP infected jejunal and jejuno-ileal tissues had more pathway activations and of higher magnitude than MAA. Interestingly, several key pathways had reversed states of activation and/or repression which maybe hypothesized as evidence of pathogenic differences between MAP and MAA host response.

We found it advantageous to define an early stage (30 and 60 minutes post infection) and late stage (480 and 720 minutes post infection) host immune response to assess which pathway/GO responses may be related to the pathogen invasion/evasion (early stage) and which may be associated with an effective or ineffective host defensive response (late stage). Table 3(A) lists the biological processes found to have a reversed state of activation and/or repression for pathways and GO immune categories organized by early and late stages. The pathways and GO categories found to be unique to the MAP condition or the MAA condition are listed in Table 3(B) and 3(C) respectively and are also organized into early and late stage responses. For example, some of the early stage reversed pathways include the B cell receptor signaling, Focal adhesion and Adherens junction pathways. Unique MAP pathway/GO activations include Cytokine-cytokine receptor interaction, mTOR signaling, and Interferon-gamma-mediated signaling pathway. Unique MAA pathway/GO activations include Regulation of actin cytoskeleton, Phagosome, and Regulation of T-helper 1 type immune response. Model interrogation results for pathways listed on Table 3 are provided in S12 Table.

**Table 3:** Early and Late Stage Pathway and Gene Ontologies Having Reversed and unique Perturbation States during MAP and MAA Interaction.

**Table 3(A) Early and Late Stage Pathway and Gene Ontologies Having Reversed Perturbation States**				
**Early Stage Reversed States [30–60 minutes post infection (p.i.)]**	**DBGGA Zscore**			
	**MAP**	**MAA**	**MAP**	**MAA**
***Pathways Reversed Activated or Repressed***	**30 min.**	**30 min.**	**60 min.**	**60 min.**
Adherens junction	-2.03	2.07	0.00	0.00
Focal adhesion	-1.98	1.42	0.00	0.25
B cell receptor signaling pathway	2.31	-2.36	0.00	1.26
TGF-beta signaling pathway	2.95	-1.57	0.00	0.88
Calcium signaling pathway	3.15	-1.76	0.00	0.00
Gap junction	3.20	-2.31	0.00	-0.11
**GO Categories Reversed Activated or Repressed**				
complement activation	2.96	-1.83	0.73	1.65
negative regulation of immune effector process	2.13	-2.07	0.00	-0.94
negative regulation of immune response	2.10	-3.45	0.76	1.08
regulation of interferon-beta production	4.58	-2.39	0.00	-2.34
regulation of type 2 immune response	2.95	-2.24	0.00	-0.45
epithelial cell proliferation	-2.90	2.49	0.00	1.49
***Late Stage Reversed States (480–720 minutes p*.*i*.*)***	**DBGGA Zscore**			
	**MAP**	**MAA**	**MAP**	**MAA**
**Pathways Activated or Repressed**	**480 min.**	**480 min.**	**720 min.**	**720 min.**
Regulation of actin cytoskeleton	-2.15	-0.01	3.64	-2.81
Melanogenesis	2.97	0.00	3.56	-2.93
Hematopoietic cell lineage	0.53	0.00	3.40	-2.15
Complement and coagulation cascades	1.78	0.13	3.35	-3.25
Chemokine signaling pathway	-0.55	0.00	3.17	-3.32
Axon guidance	0.73	0.00	2.99	-3.52
Cytokine-cytokine receptor interaction	0.48	0.00	2.92	-2.35
Tight junction	0.94	0.48	2.89	-2.41
NOD-like receptor signaling pathway	-0.62	-0.18	2.74	-2.87
Cytosolic DNA-sensing pathway	2.28	0.00	2.64	-2.43
Jak-STAT signaling pathway	-0.19	0.00	2.59	-2.82
Gap junction	-0.18	0.00	-1.99	2.57
B cell receptor signaling pathway	-0.28	-0.38	-2.02	2.61
Phosphatidylinositol signaling system	-1.62	0.00	-2.11	3.64
Osteoclast differentiation	-0.37	0.33	-2.96	2.50
ErbB signaling pathway	-0.28	-0.25	-2.97	2.92
Toll-like receptor signaling pathway	-0.43	0.00	-3.04	2.12
RIG-I-like receptor signaling pathway	1.51	0.00	-3.13	2.68
Fc gamma R-mediated phagocytosis	-1.34	-0.01	2.03	-1.95
**GO Categories Reversed Activated or Repressed**				
phagocytosis	2.00	2.00	-2.34	3.17
chemokine biosynthetic process	-2.14	0.72	3.59	-4.09
chemokine receptor activity	0.00	0.00	3.20	-1.87
complement activation	-0.16	0.53	1.65	-2.43
leukocyte homeostasis	2.16	0.00	2.49	-3.36
positive regulation of defense response to virus by host	1.18	0.00	-1.92	3.06
positive regulation of humoral immune response	-2.12	0.73	-3.00	3.21
positive regulation of immunoglobulin mediated immune response	-1.75	0.13	-1.98	2.60
**Table 3(B) Early and Late Stage Pathway and Gene Ontologies Unique to MAP Condition**				
**Eearly Stage MAP Condition Unique (30–60 minutes p.i.)**	**DBGGA Zscore**			
** **	**MAP**	**MAA**	**MAP**	**MAA**
**Unique Pathway Activated or Repressed**	**30 min.**	**30 min.**	**60 min.**	**60 min.**
Complement and coagulation cascades	3.13	1.17	0.00	0.75
ABC transporters	3.11	-1.06	0.00	1.00
Cytosolic DNA-sensing pathway	2.85	1.51	0.00	1.25
SNARE interactions in vesicular transport	2.83	-1.57	0.00	1.55
Cytokine-cytokine receptor interaction	2.50	0.86	0.00	0.03
Notch signaling pathway	2.32	1.40	0.00	-0.05
Hematopoietic cell lineage	2.25	1.41	0.00	-0.35
Adipocytokine signaling pathway	2.24	-0.93	0.00	0.00
mTOR signaling pathway	2.15	-0.59	0.00	-1.32
ErbB signaling pathway	2.12	-0.42	0.00	0.05
Toll-like receptor signaling pathway	2.12	1.68	0.00	1.23
Aldosterone-regulated sodium reabsorption	-2.20	-1.40	0.00	-0.39
Proteasome	-2.24	-1.75	0.00	-0.88
Leukocyte transendothelial migration	-2.27	-1.88	0.00	0.91
Protein processing in endoplasmic reticulum	-2.56	-1.90	0.00	-0.87
Vasopressin-regulated water reabsorption	-2.73	-1.58	0.00	0.00
Phosphatidylinositol signaling system	-2.82	-1.44	0.00	-1.00
Tight junction	-2.93	-1.89	0.00	-0.67
**GO Unique Activation and Repression**				
interferon-gamma-mediated signaling pathway	3.25	-1.46	0.00	1.39
innate immune response in mucosa	3.56	0.00	0.00	0.00
antibacterial humoral response	3.52	1.86	0.34	-0.75
regulation of immunoglobulin mediated immune response	3.16	1.64	-0.22	1.65
neutrophil mediated immunity	3.07	-1.09	0.00	0.89
adaptive immune response	3.01	1.00	0.86	1.31
complement activation	2.96	-1.83	0.73	1.65
positive regulation of defense response to virus by host	2.92	1.64	-0.24	1.66
myeloid leukocyte mediated immunity	2.65	-0.97	0.00	-0.40
chemokine receptor activity	2.62	-0.53	0.00	-0.72
positive regulation of innate immune response	2.31	0.22	0.00	-0.14
leukocyte mediated immunity	2.29	-0.62	0.00	-0.42
complement activation, classical pathway	2.22	-1.29	1.02	1.84
T cell mediated immunity	2.18	1.35	-0.85	-0.31
adaptive immune response based on somatic recombination of immune receptors built from immunoglobulin superfamily domains	2.14	0.83	-0.17	0.62
regulation of adaptive immune response	2.14	1.36	-0.52	-0.19
chemokine biosynthetic process	2.13	1.18	0.00	2.28
cell-cell adhesion mediated by integrin	-2.43	1.58	0.00	0.00
**Late Stage MAP Condition Unique (480–720 minutes p.i.)**	**DBGGA Zscore**			
	**MAP**	**MAA**	**MAP**	**MAA**
**Unique Pathway Activated or Repressed**	**480 min.**	**480 min.**	**720 min.**	**720 min.**
ECM-receptor interaction	1.97	0.00	3.17	-1.72
Vasopressin-regulated water reabsorption	2.66	0.00	2.82	1.89
Progesterone-mediated oocyte maturation	0.64	0.00	2.73	1.89
Proximal tubule bicarbonate reclamation	1.05	0.00	2.71	1.62
Cell adhesion molecules (CAMs)	1.22	0.00	2.60	1.83
ABC transporters	0.95	0.00	2.27	1.76
Renin-angiotensin system	-0.58	0.00	-2.03	-0.09
mTOR signaling pathway	1.07	-0.32	-3.20	-1.92
**GO Unique Activation and Repression**				
chemokine receptor activity	0.00	0.00	3.20	-1.87
phagocytosis, engulfment	0.00	0.00	3.04	-1.74
negative regulation of immune effector process	0.84	0.00	3.04	1.19
immune response-regulating cell surface receptor signaling pathway	0.00	-0.96	2.59	1.67
innate immune response	1.75	0.00	2.51	1.91
negative regulation of immune response	-1.49	-0.37	2.42	1.46
lymphocyte activation involved in immune response	1.48	0.00	2.36	0.72
regulation of type 2 immune response	0.00	0.00	2.32	1.57
myeloid cell activation involved in immune response	-0.60	0.24	2.23	1.94
adaptive immune response based on somatic recombination of immune receptors built from immunoglobulin superfamily domains	0.00	0.00	2.18	1.72
natural killer cell mediated immunity	0.59	0.00	-2.00	1.06
MyD88-dependent toll-like receptor signaling pathway	0.86	0.00	-2.03	1.09
regulation of T-helper 1 type immune response	0.00	0.00	-2.40	1.55
neutrophil mediated immunity	2.07	0.00	-2.94	-1.37
humoral immune response mediated by circulating immunoglobulin	2.10	0.38	-3.46	1.23
**Table 3(C) Early and Late Stage Pathway and Gene Ontologies Unique to MAA Condition**				
Eearly Stage MAA Condition Unique (30–60 minutes p.i.*)	**DBGGA Zscore**			
** **	**MAA**	**MAP**	**MAA**	**MAP**
**Unique Pathway Activated or Repressed**	**30 min.**	**30 min.**	**60 min.**	**60 min.**
Regulation of actin cytoskeleton	2.26	-1.84	0.53	0.00
Neuroactive ligand-receptor interaction	2.04	1.83	0.33	0.00
VEGF signaling pathway	-1.96	1.51	1.18	0.00
Fc epsilon RI signaling pathway	-2.06	1.27	1.28	0.00
Phagosome	-2.53	-1.88	0.19	0.00
Antigen processing and presentation	-2.14	1.65	-0.57	0.00
**GO Unique Activation and Repression**				
cytokine production involved in immune response	3.14	1.86	-1.30	-0.20
regulation of innate immune response	2.50	1.81	1.53	0.00
regulation of T-helper 1 type immune response	2.18	1.49	-0.61	-0.77
regulation of adaptive immune response based on somatic recombination of immune receptors built from immunoglobulin superfamily domains	2.10	1.71	-0.48	-0.52
type 2 immune response	-2.26	1.11	-0.35	0.00
T cell costimulation	2.33	0.05	-0.78	0.00
interleukin-12 production	2.19	1.55	1.19	0.00
negative regulation of JAK-STAT cascade	-2.95	-1.21	-0.23	-0.22
Late Stage MAA Condition Unique (480–720 minutes p.i.*)	**DBGGA Zscore**			
**Unique Pathway Activated or Repressed**	**MAA**	**MAP**	**MAA**	**MAP**
	**480 min.**	**480 min.**	**720 min.**	**720 min.**
Fc epsilon RI signaling pathway	-0.70	0.00	2.55	-1.60
p53 signaling pathway	0.00	-0.09	2.46	-1.67
Notch signaling pathway	0.00	-0.75	-2.67	-1.67
**GO Unique Activation and Repression**				
positive regulation of production of molecular mediator of immune response	-0.41	0.00	3.00	0.63
regulation of cytokine production involved in immune response	-0.24	-0.64	2.28	1.63
immune effector process	0.00	-0.34	1.98	0.93
positive regulation of myeloid leukocyte cytokine production involved in immune response	0.00	1.02	-2.25	1.31

(A) Pathways and GO immune categories showing a reversal in states of activation or repression between MAP and MAA condition and separated by early and late stages. (B) The pathways and GO categories found to be unique to the MAP condition. (C) The pathways and GO categories found to be unique to the MAA condition separated into early and late stages.

Several of the DBGGA scored Gene Ontology (GO) categories also identified contrasting differences between MAP and MAA host response during the early stage (30–60 min.). Focusing on several top scoring immune related GO categories, the MAP-MAA comparative heatmap (Fig 4) indicates a much stronger perturbation of several GO gene sets. The MAP host response had high activation of processes associated with regulation of interferon-beta production, leukocyte homeostasis, chemokine biosynthetic process, and innate immune response in mucosa. Highly repressed processes included positive regulation of endocytosis, cell-cell adhesion mediated by integrin, and negative regulation of defense response to virus. Three significant differences observed between MAP and MAA response were the reversed states of the processes of Epithelial cell proliferation, Regulation of interferon-beta production, and Regulation of type 2 immune response. Such comparisons provide compelling evidence suggesting that the virulence factors of MAP are manipulating epithelial cell proliferation, interferon production, and immune responses immediately after MAP infection and are possibly associated with its persistence in the host. The detailed analysis of several of the most interesting MAP/MAA pathway and GO perturbation pathogenic differences is presented in S2 File.

**Fig 5:**
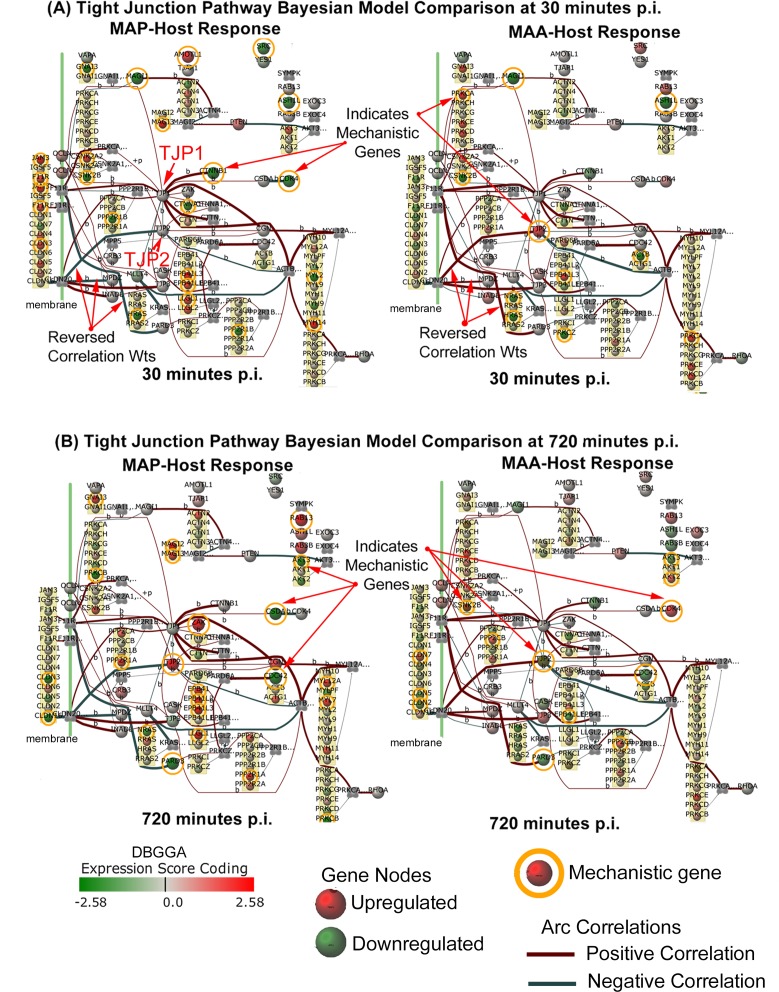
(A) The MAP-MAA condition comparison of the Tight Junction pathway Bayesian models with the state of gene expression and mechanistic genes shown for the 30 min post infection time point. (A) The same MAP-MAA condition comparison with the state of gene expression and mechanistic genes shown for the 30 min post infection time point. The legend indicates the meaning of node colors (representing a gene expression level) and arcs between connecting genes.

**Fig 6:**
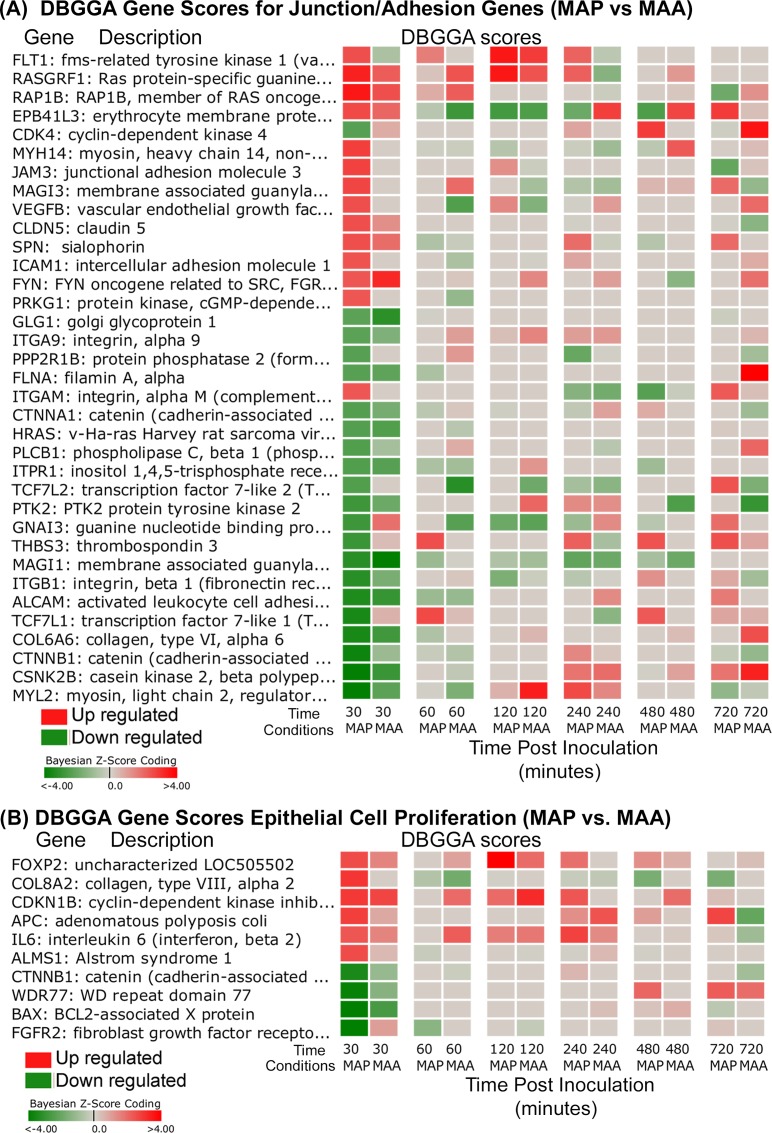
(A) Heatmap of MAP and MAA condition comparison of DBGGA zscores for mechanistic genes in the Gap Junction, Adherens Junction, Tight Junction, and Focal Adhesion junction pathways. (B) Heatmap MAP and MAA condition comparison of DBGGA zscores for mechanistic genes in the Gene Ontology category “Epithelial cell proliferation”.

The interpretation of pathway/GO pertubations, described in S2 File, suggest that MAP has a strong influence on the mucosal barrier and cell adhesion processes. These observed responses suggest that the MAP host invasion may be modulating critical cell adhesion processes in a complex manner distinctly different than MAA. Prior studies have shown that up regulation (increased gene expression) of the junction/adhesion pathways may lead to strengthening the intestinal mucosal barrier while down regulation (disrupted gene expression) may result in weakening of this immune barrier. Reported previously [14], the MAP infection caused a marked decrease in the Trans-Epithelial Resistance (TER) of an *in vitro* model of polarized epithelial cells, suggesting that increased permeability of *in vivo* host intestinal epithelium may facilitate bacterial invasion through the intestinal epithelium. The mucosal barrier has three major components, the mucus layer, the epithelial glycocalyx and the surface epithelium itself, whose integrity largely depends on Tight Junction function [24]. The Tight Junction would appear to play a major role in the pathogenic difference between the MAP and MAA host response. As described in S2 File, we developed a method to interrogate pathway Bayesian network models to identify mechanistic genes which takes into consideration the strength of gene-to-gene correlations and the state of perturbation. The Tight Junction pathway was found to have several significant mechanistic genes and gene-to-gene correlation differences as graphically illustrated in the network models of Fig 5A and 5B. Detailed comparison of modeling results identified the common and most divergent mechanistic genes as listed in Tables 4–6. The most striking candidate mechanistic relationships that define distinct MAP/MAA differences are CLDN7→TJP1, CLDN7→TJP2, CLDN7→TJP3, CDK4→CSDA, ACTB→MYL2, ACTG1→MYL2, ACTN1→MAGI3, CLDN7→MPDZ, CLDN7→INADL, JAM3→TJP1, and ACTN3→MAGI3 (where → ≡interaction/association). Although we identify these differences in more detail in S2 File, an important example is the strong down regulation of CLDN7 (claudin 7) by MAP. CLDN7 encodes an integral membrane protein and component of the tight junction strands that serve as a physical barrier to prevent solutes and water from passing freely through the paracellular space between epithelial or endothelial cell sheets. Prior research showed that CLDN7 deficient mice severely disrupted the integrity of the mucosal/epithelial barrier [25]. Interestingly, in the late stage, the Tight Junction pathway for the MAP condition becomes strongly activated. In contrast, the MAA early stage Tight Junction pathway host response was not significantly perturbed, suggesting that MAA may not be as efficient as MAP to cross the epithelial barrier. Unexpectedly, by the late stage, the MAA condition Tight Junction perturbation did become significantly repressed (Table 3(A) late stage). The junction and cell adhesion related mechanistic gene DBGGA Z-scores are shown in the MAP-MAA comparative heatmap of Fig 6A. In the early stage (30 minutes post infection), MAP has a higher number of significant down regulated genes in comparison to MAA. While in the late stage most junction/adhesion related genes are less expressive in both conditions.

**Table 4:** Common MAP and MAA Arcs and Mechanistic Genes in Tight Junction Pathway.

Parent Gene	Parent Gene Description	Child cause	Childgene	MAP Norm. Arc Wt.	MAA Norm. Arc Wt.	Abs. Val. Difference	Correl. Direction	MAP 1st Mechanistic Time	MAP Parent Gene Score	MAP Child Gene Score	MAA 1st Mechanistic Time	MAA Parent Gene Score	MAA Child Gene Score
PARD3	par-3 partitioning defective 3 homolog (C. elegans)	JAM3	junctional adhesion molecule 3	0.27	0.56	0.29	positive	t = 30	0	2.62	t = 720	-2.9	0
ACTB	actin, beta	MYL12A	myosin, light chain 12A, regulatory, non-sarcomeric	0.2	0.38	0.18	positive	t = 720	0	2.12	t = 30	-3.72	0
CLDN7	claudin 7	MPDZ	multiple PDZ domain protein	-0.42	-0.2	0.33	negative	t = 60	-3.17	0	T = 480	2.4	0.81
TJP1	tight junction protein 1	ACTG1	actin, gamma 1	0.39	0.26	0.13	positive	t = 60	0	2.04	t = 60	0.74	2.57
ACTG1	actin, gamma 1	MYH9	myosin, heavy chain 9, non-muscle	0.56	0.45	0.11	positive	t = 60	2.04	-1.37	t = 60	2.57	-1.55
CDC42	cell division cycle 42 (GTP binding protein, 25kDa)	PARD6B	par-6 partitioning defective 6 homolog beta (C. elegans)	0.74	0.44	0.3	positive	t = 720	-3.53	-0.81	t = 240	2.53	0
CLDN7	claudin 7	TJP3	tight junction protein 3 (zona occludens 3)	0.6	0.23	0.37	positive	t = 60	-3.17	-1.81	t = 480	2.4	0
CDK4	cyclin-dependent kinase 4	CSDA	cold shock domain protein A	-0.36	-0.2	0.16	negative	t = 30	-2.37	-0.11	t = 240	0	-2.7
CSNK2B	casein kinase 2, beta polypeptide	no child node		0	0	0	positive	t = 30	-4.07	0	t = 30	-2.94	0
CTNNA1	catenin (cadherin-associated protein), alpha 1, 102kDa	no child node		0	00		positive	t = 30	-2.27	0	t = 240	2.06	0
AKT2	v-akt murine thymoma viral oncogene homolog 2	no child node		0	0	0	positive	t = 60	2.08	0	t = 720	2.47	0
PRKCZ	protein kinase C, zeta	no child node		0	0	0	positive	t = 240	2.72	0	t = 30	-2.75	0
EPB41L1	erythrocyte membrane protein band 4.1-like 1	no child node		0	0	0	positive	t = 30	2.03	0	t = 240	-2.36	0

Tight Junction Pathway Bayesian Model Interrogation Results of Gene-to-Gene Relationships and Mechanistic Genes found in common between the MAP and MAA conditions. See text for description of interrogation methods.

**Table 5:** Unique MAP Arcs and Mechanistic Genes in Tight Junction Pathway.

Parent Gene	Parent Gene Description	Child Gene	Child Gene Description	MAP Norm. Arc Wt.	MAP Norm. Arc Wt.	Abs. Val. Difference	Correl. Direction	MAP 1st Mechanistic Time	MAP Parent Gene Score	MAP Child Gene Score	MAP Child Gene Score	MAP Child Gene Score	MAA Child Gene Score
ACTN1	actinin, alpha 1	MAGI3	membrane associated guanylate kinase, WW and PDZ domain containing 3	0.21	-0.03	0.24	reverse	t = 30	0.71	2.65	t = 60	0	1.99
CLDN7	claudin 7	TJP2	tight junction protein 2 (zona occludens 2)	-0.43	0.15	0.58	reverse	t = 60	-3.17	0	t = 30	-0.92	2.02
RRAS2	related RAS viral (r-ras) oncogene homolog 2	MLLT4	myeloid/lymphoid or mixed-lineage leukemia (trithorax homolog, Drosophila); translocated to, 4	-0.41	0.1	0.51	reverse	t = 60	-0.41	2.17	t = 480	-0.51	-3.1
ACTG1	MYH14	MYH14	myosin, heavy chain 14, non-muscle	0.3	0.1	0.2	positive	t = 30	-0.98	2.75	t = 60	2.57	-0.23
PRKCB	protein kinase C, beta	ACTB	actin, beta	0.27	0.1	0.17	positive	t = 720	-2.69	0	t = 30	0	-3.72
PARD3	par-3 partitioning defective 3 homolog (C. elegans)	F11R	F11 receptor	-0.46	-0.18	0.28	negative	t = 30	0	1.99	t = 720	-2.9	-1.05
TJP1	tight junction protein 1	CTNNB1	catenin (cadherin-associated protein), beta 1, 88kDa	0.48	0.3	0.18	positive	t = 30	0	-4.29		0	-1.38
CLDN7	CLDN7	TJP1	tight junction protein 1	0.23	0.03	0.2	positive	t = 60	-3.17	0	t = 480	2.4	-0.69
CLDN7	CLDN7	CLDN7	InaD-like (Drosophila)	0.47	0.12	0.35	positive	t = 60	-3.17	0	t = 480	2.4	0
ACTG1	actin, gamma 1	MYL9	myosin, light chain 9, regulatory	0.3	0.13	0.17	positive	t = 60	2.04	0	t = 60	2.57	0
ACTN3	actinin, alpha 3	MAGI2	membrane-associated guanylate kinase, WW and PDZ domain-containing protein 2-like	0.21	0.07	0.14	positive	t = 120	0	3.05	t = 120	0	2.98
EPB41L3	erythrocyte membrane protein band 4.1-like 3	CASK	calcium/calmodulin-dependent serine protein kinase (MAGUK family)	0.21	0.04	0.17	positive	t = 30	2.62	0	t = 30	2.02	0.6
ACTG1	actin, gamma 1	MYL7	myosin, light chain 7, regulatory	0.38	0.17	0.21	positive	t = 60	2.04	-0.62	t = 60	2.57	-0.44
PARD3	par-3 partitioning defective 3 homolog (C. elegans)	IGSF5	immunoglobulin superfamily, member 5	0.23	0.11	0.12	positive	t = 720	-2.71	0	t = 720	-2.9	-0.53
JAM3	junctional adhesion molecule 3	TJP1	tight junction protein 1	0.29	0	0.29	positive	t = 30	2.62	0		0	0
PRKCB	protein kinase C, beta	IGSF5	immunoglobulin superfamily, member 5	-0.24	-0.13	0.11	negative	t = 720	-2.60	0		0.99	-0.53
ACTN3	actinin, alpha 3	MAGI3	membrane associated guanylate kinase, WW and PDZ domain containing 3	-0.21	0.04	0.25	reverse	t = 30	0	2.65	t = 60	0	1.99
PPP2R1A	protein phosphatase 2, regulatory subunit A, alpha	no child node		0	0	0	positive	t = 720	2.14	0		1.18	0

Tight Junction Pathway Bayesian Model Interrogation Results found unique to the MAP condition.

**Table 6:** Unique MAA Arcs and Mechanistic Genes in Tight Junction Pathway.

Parent Gene	Parent Gene Description	Child Gene	Child Gene Description	MAANorm. Arc Wt.	MAA Norm. Arc Wt.	Abs. Val. Difference	Correl. Direction	MAA 1st Mechanistic Time	MAA Parent Gene Score	MAA Child Gene Score	MAP 1st Mechanistic Time	MAP parent Gene Score	MAA Child Gene Score
ACTB	actin, beta	MYL7	myosin, light chain 7, regulatory	0.23	-0.01	0.24	reverse	t = 30	-3.72	0	t = 720	0	2.11
ACTG1	actin, gamma 1	TJP2	tight junction protein 2 (zona occludens 2)	-0.45	-0.17	0.28	negative	t = 30	0.03	2.02	t = 60	2.04	0
ACTB	actin, beta	MYH14	myosin, heavy chain 14, non-muscle	0.28	0.16	0.12	positive	t = 30	-3.72	0		0	0
ACTB	actin, beta	MYH9	myosin, heavy chain 9, non-muscle	0.58	0.42	0.16	negative	t = 30	-3.72	0		0	0
ACTB	actin, beta	MYH1	myosin, heavy chain 1, skeletal muscle, adult	-0.29	-0.44	0.15	negative	t = 30	-3.72	0		0	0

Tight Junction Pathway Bayesian Model Interrogation Results found unique to the MAA condition.

The Gene Ontology (GO) term “cell-cell adhesion mediated by integrin” was also significantly repressed at the early stage for the MAP condition. Cell adhesion serves to facilitate trafficking and migration of T lymphocytes into sites of inflammation, movement of lymphocytes within the rich environment found in extravascular tissue, and the physical interaction between antigen-reactive T cells and antigen-presenting cells that is required for efficient T-cell activation [26]. The repressed junction/adhesion related pathways and their associated genes suggest that MAP may disrupt T lymphocyte recruitment, which helps explain the lack of chronic inflammation observed in the MAP infected ileal loops and may further subvert mucosal healing [14]. It has been proposed by others [27] that a bacterial survival mechanism in mucosal epithelial cells is for the bacteria to hijack integrin-linked kinase to stabilize focal adhesions and block cell detachment of infected cells. The rapid turnover and exfoliation of mucosal epithelial cells provides an innate defense system against bacterial infection. The significant repression and reversed state of the GO category, “Epithelial cell proliferation”, (Table 3(A) early stage) provides evidence of MAP interference with this important host defensive process and suggests that bacteria such as MAP may be able to subvert this immune defense mechanism and colonize the epithelium more efficiently to survive. Furthermore, for MAA, the GO category “Epithelial cell proliferation”, host response was strongly activated, suggesting a strengthening of the mucosal/epithelial barrier and perhaps decreasing the efficiency of MAA invasion. Fig 6(B) shows a heatmap of the DBGGA gene scores for the Epithelial cell proliferation gene set. At 30 minutes post infection, the MAP condition shows both strong down and up regulation of genes in comparison to the low expression values in MAA.

The Toll-like receptor pathway was strongly activated in MAP but only modestly activated in MAA in the early stage. For the MAP condition, the early stage pathway activation was dominated by TLR3 up regulation, but this becomes insignificantly expressed in the late stage. Interestingly, in the MAA condition, TLR3 was only significantly up regulated at 240 minutes post infection perhaps suggesting a delayed TLR response. However, for the MAA early stage condition, TLR9 was significantly up regulated, but insignificant in the late stage. In contrast, TLR9 was not expressed in the MAP condition until it became significantly down regulated in the late stage (720 minutes post infection). Interestingly, TLR2, TLR4, TLR5 and TLR6 were not significantly expressed in either condition, but the gene CD14, which acts as a co-receptor with TLR4 for the detection of bacterial lipopolysaccharide (LPS) was significantly expressed in both MAP and MAA infected hosts (early stage) and became insignificantly expressed in the late stage. Numerous mycobacterial studies have revealed that TLR2 is involved in innate recognition and responses in the innate immune cells [28], which appears to be contradictory to that found in this *in vivo* study in which TLR3 and TLR9 were the only significantly up regulated TLRs for MAP and MAA respectively.

TLR3 (up regulated in MAP) plays a fundamental role in pathogen recognition and should mediate the production of proinflammatory cytokines necessary for the development of an effective immune response. TLR3 activation should be inducing the activation of IRF3 which, in turn, should induce the production of type I interferons (IFNs). However, IRF3 was significantly down regulated in both MAP and MAA conditions in the early stage and there was insignificant expression of any type I IFNs or type II IFNs. Moreover, the GO categories for Regulation of interferon-beta (IFN-β) and Interferon-gamma (IFN-γ)-mediated signaling were significantly activated in MAP condition, but were both repressed in the MAA condition. Interestingly, in the MAP condition (early and late stage), the chemokine CXCL9 is up regulated, but down regulated in MAA condition (early stage). CXCL9 is known to be a T-cell chemoattractant that is normally induced by IFN-γ. However, the MAP infected host did have several up regulated cytokines that included IL1β, IL4, CCL25, CX3CL1, CCL20, IL7, IL18, CXCL9, and IL10 primarily all during the late stage. Interestingly, some of these cytokines are predicted to be regulators of immune outcome during mathematical modeling of MAP infection, and also regulators of infection outcomes in clinical cases in mycobacterial disease [29–31].

TLR9 (up regulated in MAA only) is preferentially expressed in immune cell rich tissues from cells such as B lymphocytes, monocytes, natural killer (NK) cells, and plasmacytoid dendritic cells. TLR9 is activated by microbial DNA containing unmethylated CpG motifs. MAP is proposed to evade host immunity by altering the TLR9 signaling [31]. TLR9 signals lead to the activation of cells initiating pro-inflammatory reactions in the production of cytokines such as type-I interferon and IL-12. Interestingly, IFN-β was insignificantly expressed in the MAA infected host, but the GO category “Regulation of cytokine production involved in immune response” was significantly activated suggesting MAA was activating some cytokine response. However, the only cytokines significantly up regulated in the MAA infected host were TNF, CCL5, IL18, IL5, IL12β, IL4, and CXCL9. TNF is associated with apoptosis. In both the MAP and MAA infected host, there was activation of apoptosis only in the late stage. CCL5 plays an active role in recruiting leukocytes into inflammatory sites. IL5 stimulates B cell growth and increases immunoglobulin secretion. IL18 together with IL-12 induces cell-mediated immunity following infection and exposure to microbial products like LPS. TLR9 has been reported to be essential for the efficient control of MAA infection, however it could be independent of Th1 type cytokine induction [31]. S13 Table shows the Bayesian model interrogation results of gene-to-gene relationships and Mechanistic genes for Toll-like Receptor Pathway for genes common between the MAP and MAA conditions (A), unique to the MAP condition (B), and unique to the MAA condition (C).

Interestingly, it has been reported in some studies that mycobacterial evasion may involve increased secretion of IL10 rather than IL12 by dendritic cells. Secretion of IL10 will favor the activation of a Th2 response which is incapable of destroying intracellular pathogens. The MAP infected host did, in fact, have increased IL10 expression and strong activation of the GO category “Regulation of type 2 immune response” and the strong repression of the category “Regulation of T-helper 1 type immune response”, whereas MAA favored IL12B expression combined with the activation of the “Regulation of T-helper 1 type immune response” and the repression of “Type 2 immune response”. Another speculated mycobacterial evasion method involves the stimulation of antigen presenting cells with mycobacterial lipoprotein that results in decreased major histocompatibility class II complex (MHCII) expression [32] where MHCII molecules are critical for the initiation of the antigen-specific immune response. In the MAP infected host, the MHCII gene BOLA-DMA was significantly down regulated while in the MAA infected host, the MHCII gene HLA-DOA was significantly up regulated. MHCII gene upregulation has been correlated with the production of TNF and IL12 that may lead to containment of MAA infection [33].

Mycobacteria are usually internalized by macrophages via phagocytosis and reside in phagosomes to help escape destruction reportedly by inhibiting phagosome/lysosome maturation. For the MAP infected host, the DBGGA analysis found the GO category “Phagocytosis” to be activated briefly at 240 minutes post infection but becoming significantly repressed in the late stage, while in the MAA infected host it remained strongly activated. Examining the mechanistic genes of “Phagocytosis”, the main differences are significant up regulation of HMGB1, CORO1A, and CSK for the MAA infected host, early stage, while in the late stage the dominant genes that induced the repressed state of “Phagocytosis” for MAP condition were PIP5K1C, HMGB1, and CORO1A. The gene PIP5K1C encodes the protein, phosphatidylinositol 4-phosphate 5-kinase type-1 gamma involved in a number of cellular functions, but most relevant is that PIP5K1A is required for the regulation of actin remodeling in phagocytosis. The role of actin cytoskeleton remodeling during particle internalization is well established, but its role during the later stages of phagosome maturation (phagosome/lysosome fusion) remains largely unknown. The disruption of this gene may be involved in the MAP pathogenicity by impeding phagolysosomal fusion and hence, inhibiting phagosome maturation. Further, the repressed state of the “phosphatidylinositol signaling system” in the MAP condition (early stage) provides additional evidence that MAP may disrupt this process.

Our detailed analysis (S2 File) suggests that the MAA infected host response appears to be mounting a stronger cell mediated immune response as evident by the significant activations of the B cell receptor signaling pathway and the GO categories “Regulation of T-helper 1 type immune response”, “Regulation of adaptive immune response”, “Cytokine production involved in immune response”, “Regulation of innate immune response”, “T cell costimulation”, and “IL12 production”. The overall number of unique pathway and GO category perturbations, however, are far less in the MAA condition which supports the observation that MAA is not as pathogenic as MAP. Interestingly, there are a large number of strongly perturbed pathways and GO categories that show significant reversal of activation states between MAA and MAP. For example, at the late stage, the MAA condition has 16 pathways and GO categories that become strongly repressed in comparison to the 16 activated pathway states for MAP (Table 3(A)), suggesting that the host may be struggling to stop the proliferation of MAP while for MAA, the immune response may be shutting down as the host clears the MAA bacteria.

### Discovery of Unique and Common Mechanistic Genes during MAP and MAA Infection

Pathway and GO DBGGA analysis provided a list of mechanistic genes over the time course of the MAP and/or MAA infection. A mechanistic gene is found based on how much the gene contributes to the perturbation of the pathway and/or GO term gene set. For pathways, the contribution is determined in the context of the network and the gene’s relationships to other genes in the pathway Bayesian network structure (S1 File). The GO mechanistic genes are determined by the contribution of the perturbed gene as part of the overall GO gene set modeled as a naïve Bayesian net which differs from the pathway analysis by not taking into consideration any network structure relationships. For the pathway analysis, S5 and S6 Tables lists the associated mechanistic genes filtered having a gene DBGGA |Z-score| ≥ 2.24 for the MAP and MAA conditions, respectively. The GO gene DBGGA Z-Scores are provided in S9 and S10 Tables for the MAP and MAA conditions respectively.

**Fig 7:**
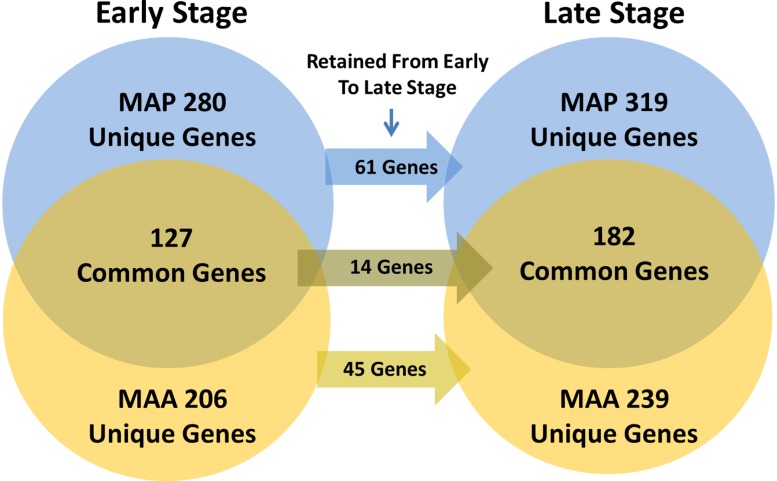
Venn diagram comparison of the number of unique and common mechanistic genes found for the MAP and MAA conditions. Only the mechanistic genes from the pathways and GO terms listed in Table 3 were used in creating this diagram.

For each of the significantly perturbed pathways and GO categories listed in Table 3, we determined which mechanistic genes were in common between the MAP and MAA conditions and which were unique at both the early and late stage post infection. The results of this comparative analysis are illustrated in the Venn diagrams of Fig 7 with details provided in S11 Table. We found that MAP and MAA conditions had 127 perturbed mechanistic genes in common in the early stage, while MAP had 280 uniquely perturbed mechanistic genes and MAA had 206. As the course of infection transitioned to the late stage, there were 182 genes in common, while MAP had 319 and MAA had 239 uniquely perturbed. The transition from early to late stage retained only a small percentage of the same genes which suggests that the host immune response patterns diverge considerably between conditions and over time. Interestingly, some of the pathways with the highest number of mechanistic genes in common between MAP and MAA condition during the early stage were ECM-receptor interactions, Hematopoietic cell linage, Tight junction, Phagosome, Toll-like receptor signaling, RIG-I-like receptor signaling, and Chemokine signaling, along with the GO terms Epithelial cell proliferation and MyD88-dependent toll-like receptor. In the late stage, the pathways having the highest number of mechanistic genes in common included such pathways as Cell adhesion molecules, Cytokine-cytokine receptor interactions, Fc gamma R-mediated phagocytosis, Jak-STAT signaling, Phosphatidylinositol signaling system, Axon guidance, Adipocytokine signaling, and ErbB signaling pathway, along with the GO terms Innate immune response in mucosa, T cell co-stimulation, and Interferon-gamma-mediated signaling. Probably the most interesting pathways that were found unique to MAP condition during the early stage having the highest number of mechanistic genes included ABC transporters, ECM-receptor interaction, and Tight junction whereas in the MAA condition the early stage pathways with higher numbers of mechanistic genes were Gap junction, Phagosome, Axon guidance, and Fc epsilon RI signaling pathway. In the late stage, the pathways that had the highest number of mechanistic genes in the MAP condition included such pathways as Hematopoietic cell lineage, Calcium signaling, SNARE interactions in vesicular transport, Toll-like receptor signaling, mTOR signaling, and Epithelial cell proliferation whereas in the MAA condition the interesting pathways included Gap junction, Notch signaling, Leukocyte transendothelial migration, Adipocytokine signaling, VEGF signaling, and Adherens junction. These results reveal that perturbation of genes and cellular pathways during MAP infection was focused on host evasion facilitating persistent infection. However, MAA infection resulted in the activation of molecular pathways that are involved with containment of infection and resulting in transient infection.

## Discussion

A key role of intestinal mucosal epithelium is barrier function, which prevents colonization or invasion of the host by foreign microorganisms. Transmission of MAP or MAA in young calves usually occurs via feeding on milk contaminated with these pathogens. The bacterium is also shed in the feces; therefore fecal-oral route is another mode of transmission. Here, in this study, ileal loop were directly inoculated with MAP or MAA to dissect the changes in the transcription of genes in the intestinal mucosa. Intestinal transcription responses usually do not alter much due to the presence of commensal microbiota. However, when pathogens come in contact with the epithelial surface of the ileal mucosa, there is a change in the host gene expression. The alteration in the host gene expression may depend on the type of pathogen, pathogenicity, virulence factors, and replication rate of the pathogen among several other factors. The pathogenesis of MAP infection in ruminants and other species shows typical Th1/Th2 type of immune profile [34–37]. Recently, a study showed that the histopathology of the cutaneous delayed type hypersensitivity response were almost similar as the stage of intestinal pathology of MAP infection [38]. Moreover, this also matched well with the systemic humoral and cellular immunity. Strong gut-associated immune responses participate in the elimination of pathogenic microorganisms, while immune tolerance prevents harmful reactions against the gut flora and food antigens. We have utilized the neonatal calf ligated jejuno-ileal loop model to study the sequential changes in the host intestine after infection with MAP and MAA. In the ligated ileal loop model, one can only humanely conduct the experiment for about 12 hours under general anesthesia. Thus, we did not attempt to go beyond this time period. There could be concerns that concentration of bacteria used in this study to inoculate ileal loop is higher than the estimated concentration of MAP in natural conditions, in the farms, where lower concentrations of MAP are able to start an infection. However, the initial infection dose is undetermined under farm environmental conditions, and may require more time to establish an infection. Earlier studies have shown that animals orally inoculated with 5×10^9^ CFU given on 2 consecutive days were able to establish infection [39]. Though, we inoculated 3x10^9^ CFU, the invasion rate of the MAP was very low (results of our invasion of bacteria in the mucosa). Moreover, in our study the aim was to test the acute dose responses during the early establishment phase of infection. One would expect very similar host gene expression responses when genetically similar pathogens are introduced to a host species, however this prediction was not true in the case of MAP vs. MAA enteric infection of cattle. Though the pathogens are >95% genetically similar, MAP causes a severe chronic granulomatous enteritis in cattle, whereas MAA infection is normally rapidly cleared [40]. Thus, the most important differences in the MAP and MAA pathogenesis are a chronic persistent infection vs transient infection respectively. The type of immune response triggered by these two very similar pathogens appears to depend, in part, on initial recognition by the innate immune system. Our results indicate that MAP causes a rapid and early perturbation of cellular components. This perturbation results in dysfunction or dysregulation of the innate immune system in comparison to MAA. When this concept is applied to natural infection with MAP, a chronic activation of innate immunity may negatively affect adaptive immune responses, contributing to a persistent infection. However, the components of the immune response are only partially dysfunctional during MAA infection and thus may lead to a transient infection with an outcome of clearance of infection.

In a recent study published by our group, the early host response was evident by the presence of MAP infected M cells and goblet cells [13]. M cells are unique among cells of the intestinal epithelium as they display a high density of ß1 integrins on their luminal surface. Integrins have affinity for the fibronectin attachment protein of mycobacteria. M cells facilitate the entry of MAP into the jejuno-ileal mucosa by forming the fibronectin bridge with host integrins [41]. Though the genes ITGß2, ITGß5, ITGA2, ITGAE, and ITGAM encoding integrins were up regulated during MAP, the integrins ITGß1 and ITGα9 were strongly down regulated. ITGß2 was significantly up regulated in the MAA infection. The expression level of ITGß2 was considerably higher in MAA infection, but MAP had two different types of down regulated integrin expression. Junctions (tight, gap and adherens) between the epithelial cells provide a mechanical barrier to the entry of infectious agents. MAP and MAA may gain entry due to the down regulation of the gene encoding tight junction protein. However, once inside the host mucosa, mycobacteria may up-regulate the tight junction related genes (Rho GTPase activating protein 10) that are involved in GTPase activation, actin remodeling and intracellular transport [42] necessary for alpha-catenin recruitment at adherens junctions and for invasion of pathogens [43]. The increased expression of these tight junction-related genes could be a reason for no increase in the invasion of MAP or MAA with the time of contact post infection.

Another important aspect of the innate response to infection is the interaction of pattern-recognition receptors (PRRs) including TLRs with PAMP molecules. Different types of leukocytes in the intestinal mucosa, i.e. dendritic cells, macrophages, eosinophils, mast cells, and natural killer cells, are localized adjacent to epithelial cells and interact with each other to maintain homeostasis [44, 45]. Our data analysis suggests that MAP may alert the host immune system by interacting with TLR3 and CD14 which are expressed on the cell surface of enterocytes and numerous cells of the immune system such as dendritic cells, B lymphocytes and NK cells. These interactions activate several signals initiating pro-inflammatory reactions that result in the production of cytokines such as IL1ß, IL4, CCL25, CX3CL1, CCL20, IL7, IL18, CXCL9, and IL10. The outcome of this interaction leads to the recruitment of leukocytes as target for intracellular replication to the site of infection. In contrast, MAA appears to interact with TLR9 binding with the intracellular TLR9 receptor inside the mast cells [46]. Apart from being a mode of entry to the host cells this interaction may also lead to cytotoxic T cell modulation as well as generation of pro-inflammatory mediators [46, 47]. The importance of innate immunity has been underscored by the studies involving mycobacterial pathogens. Our study clearly indicates that the host response starts immediately after sensing the microbial interaction with the intestinal mucosa that in turn releases signals to stimulate recruitment of pro-inflammatory leukocytes, immune cells, or both. Interactions between CD40 receptors on macrophages and CD154 (CD40L) on activated T cells are crucial for maintaining a Th1 response and activation of macrophages [48]. However, we found MAP favoring a bias toward a Th2 response whereas MAA was favoring the Th1 response. Thus, MAP apparently dampens the innate responses by affecting T cell as well as B cell activation soon after entering the host intestinal mucosa.

During MAP infection, the antigen receptors of T cells are stimulated; however, due to the apparent lack of co-stimulator molecules from APCs, further T cell activation apparently does not occur, leading to a quasi-anergic state. Interestingly, during MAA infection delivery of the second signal by the APC rescues the activated T cell from anergy, allowing it to produce the chemokines and cytokines necessary for the growth of additional T cells. These chemokines play important roles in the development, homeostasis, and function of the immune system.

The inversely expressed (up vs. down) mechanistic genes also may predict the outcome of the MAP and MAA infection. The *ATP binding cassette* was mechanistic in both MAP and MAA infections, however the subfamilies to which they belong were somewhat overlapping in MAP and MAA. In the MAP infected tissues, the *ATP binding cassette subfamily B*, *member 10*, *ATP binding cassette subfamily B*, *member 4*, *ATP binding cassette subfamily A*, *member 3*, *ATP binding cassette subfamily C*, *member 6*, *ATP binding cassette subfamily A*, *member 1 and ATP binding cassette subfamily D*, *member 3* were mechanistic, whereas in MAA infection *ATP binding cassette subfamily B*, *member 4*, *ATP binding cassette subfamily B*, *member 6*, *ATP binding cassette subfamily C*, *member 10*, *ATP binding cassette subfamily D*, *member 3* were mechanistic. The *ATP binding cassette* family of genes is involved in transmembrane movement of substances as well as transportation of cholesterol and lipids [49]. Activation of *ATP binding cassette subfamily D* in MAP during the early infection probably performs very important roles during establishment of host–pathogen interactions. In the early phase, *ATP binding cassette subfamily D* may act via host cell signaling for pathogen recognition, invasion and intracellular trafficking. During the infection establishment phase, it may play several immunomodulatory roles. During pathogen replication and persistence phases, lipid metabolism has a housekeeping role in energy homeostasis and biomembrane synthesis. Recently more emphasis has been given on the host-derived lipids in mucosal defense [50, 51]. A correlation has been shown between activation of peroxisome and mast cell maturation in allergic diseases [52].

Calcium signaling was uniquely perturbed in the MAP infected host. The dominant mechanistic genes *CALM*, *CAMK2A*,*CAMK2D*, and *CALML5* are calcium signaling molecules and prominent kinases in the central nervous system that may function in long-term potentiation and neurotransmitter release. These genes have been shown to be early responsive genes in epithelial cells [53, 54].

Chemokine ligand 5 (*CCL5*) and its receptor Chemokine receptor 4 (*CCR4*) were inversely correlated during MAP and MAA infection (up regulated in MAP, down regulated in MAA) but at different time points. CCL5 is chemotactic for T cells, eosinophils, and basophils, and plays an active role in recruiting leukocytes into inflammatory sites. *Secreted frizzed-related protein 2*, a mechanistic gene in MAP infection with its higher expression, may induce cellular regulation of apoptosis and eosinophilia [55]. The expression of these genes further supports our earlier study where we showed that the MAP infection results in the recruitment of mononuclear cells to the site of infection [13]. Usually with the help of IL-2 released by T cells, CCL5 induces the proliferation and activation of certain natural-killer cells to form CC-Chemokine-activated killer cells. However, during MAP infection, *IL-2* expression was down regulated in the late stage, which could be a reason for the absence of CC-Chemokine-activated killer cells during mycobacterial infection [56, 57]. Several genes that play significant roles in T cell or B cell activation were mechanistic during MAP infection (*CD37*, *CXCL9* and *CXCL11*). Another mechanistic gene during MAP infection was IKBKG protein (inhibitor of kappa light polypeptide gene enhancer in B cells), that interacts with two enzymes, *IKK-alpha* and *IKK-beta*, to activate nuclear factorkappa-B (*NFKB*). In contrast, the *NFKB* complex is inhibited by I-kappa-B proteins (*NFKBIB*) which is a mechanistic gene expressed during MAP infection early stage at 30 minutes post infection and was not expressed thereafter. *NFKBIB* inactivates *NFKB* by trapping it in the cytoplasm. Another proinflammatory molecule, *SERPINE 1*, was also a mechanistic gene in the MAP infection mucosal tissue. Thus, early stage activation of *NFKB* and *SERPINE 1* in MAP infection may lead to the conditions for establishing chronic infection, whereas during MAA infection, *NFKB* and *SERPINE 1* are inactivated and thus do not contribute to persistent inflammation.

In contrast to MAP infection, during MAA infection most of the mechanistic genes played role(s) in a protective immune response. Nuclear factor of activated T-cells (*NFATC1*) is a component of the DNA-binding transcription complex. Proteins belonging to this family of transcription factors have a central role in inducible gene transcription during the immune response. *NFATC1* is a mechanistic gene which was up regulated in the late stage during MAA infection, but not in MAP. It may not only provide an effective immune response by inducible expression of cytokine genes, activation and proliferation of T cells, but it also has a role in the differentiation and programmed death of T-lymphocytes as well as lymphoid and non-lymphoid cells that are involved in the capture and killing of organisms.

MAA infection perturbed the Fc epsilon RI signaling pathway soon after infection (Table 3(C)) but this pathway was not perturbed in MAP infection. Fc epsilon RI signaling is exclusive to mast cells. Mast cell driven immune responses are also linked with the activation of *NFATC1*. Activation of mast cells triggers the release of several activated molecules such as biogenic amines (histamines), proteoglycans (heparin), lipid mediators such as leukotrienes (LTC4, LTD4 and LTE4), prostaglandins (especially PGD2) and secretion of cytokines, the most important of which are IL-4 and IL-5. These mediators and cytokines along with the activation of T-cell receptors leading to T-cell proliferation and differentiation into effector cells that collectively contribute to protective responses against MAA.

MAP infection but not MAA results in activation of eosinophils via CCR3 signaling. Activated eosinophils release reactive oxygen species, which may cause tissue damage during chronic inflammatory responses.

In summary, the mechanism of infection during MAP or MAA infection may be as follow: our study revealed that perturbation of genes and cellular pathways during MAP infection was primarily focused on cell recruitment, cell proliferation and cell differentiation associated with persistent infection. Contrarily, MAA infection was related to cellular responses associated with activation of molecular pathways that produce chemicals and cytokines involved with the containment of infection. We also found that the expressions of only TLR3 and TLR9 in our *in vivo* study to be contradictory to those reported from *in vitro* studies, suggesting that the genotype of the pathogen and the site of infection in the intestinal environment plays a major role in the pathogenic mechanisms of mycobacteria. By comparing the intestinal transcriptional responses of calves infected with MAA versus MAP, unique patterns of expression were clearly evident. The number of transcripts altered were somewhat greater for MAP-infected tissue than for tissues infected with MAA. To interpret these complex data, changes in the gene expression were further analyzed by dynamic Bayesian Gene Group Activation (DBGGA) analysis and model interrogation methods. Bayesian network modeling identified mechanistic genes and gene-to-gene relationships that are involved in specific cell activation during MAP or MAA infection. Furthermore, a major difference was found in the interaction between co-stimulator molecules and T cell receptor signaling during MAP and MAA infection. Our systems biology approach not only permitted deep analysis of global gene expression during mycobacteria-induced innate immune responses, but also enabled prediction of the magnitude of the subsequent adaptive immune response and proposed new correlates of transient vs. persistent infection.

**Fig 8:**
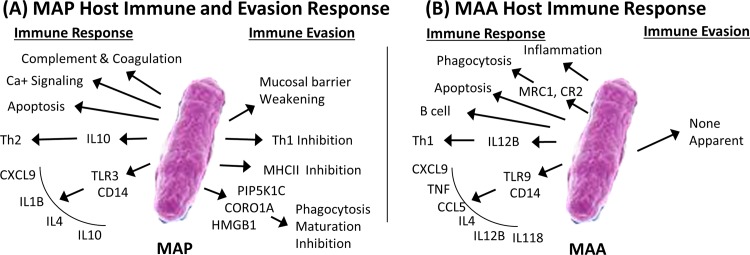
(A) Depiction of the summary conclusion for the MAP host immune and evasion responses. (B) Depiction of the summary conclusion for the MAA host immune response.

Overall, we found several distinct differences in the pathway, GO, and gene expression perturbations which allowed us to infer the mechanisms of invasion and evasion of the more persistent MAP pathogen. The important conclusions and differences between MAP and MAA are summarized in Fig 8(A) and 8(B) respectively. We propose that MAP has four immune evasion mechanisms that help prolong its survival in the host, including: 1) weakening of the mucosal immune barrier; 2) inhibition of the histocompatibility class II complex expression; 3) reduction of phago-lysosome maturation; and 4) inhibition of Th1 host immune response in favor of a Th2 response. In the transient MAA condition, the host had a strong innate immune response favoring a Th1 bias and strong phagocytic activity.

## Supporting Information

S1 File Supplemental Materials and Methods describing details used in the manuscript. (DOCX)

S2 File Detailed analysis of several of the most interesting MAP/MAA pathway and GO perturbation (DOCX)

S1 Table The complete list of Bayesian z-scored genes with associated q-values for all time points. Complete listing of all Bayesian z-scored genes with variance smoothing and outlier removal for MAP infected host. Bayesian Z-score (filtered at |zscore| ≥ 1.96 with variance smoothing, outlier removal, and false discovery rate < = 0.5
(XLSX)

S2 Table Complete listing of all Bayesian z-scored genes with variance smoothing and outlier removal for MAA infected host. (XLSX)

S3 Table Complete listing of all pathways surpassing a DBGGA |zscore| ≥ 2.24 at any one time point for the MAP infected host versus PBS. (XLSX)

S4 Table Complete listing of all pathways surpassing a DBGGA |zscore| ≥ 2.24 at any one time point for the MAA infected host versus PBS. (XLSX)

S5 Table List of all mechanistic genes (DBGGA |zscore| ≥ 2.24) found for all pathways for the MAP infected host condition. (XLSX)

S6 Table List of all mechanistic genes (DBGGA |zscore| ≥ 2.24) found for all pathways for the MAA infected host condition. (XLSX)

S7 Table Complete listing of all Gene Ontology biological processes, cellular component and molecular categories surpassing a DBGGA |zscore| ≥ 2.24 at any one time point for the MAP infected host versus PBS. (XLSX)

S8 Table Complete listing of all Gene Ontology biological processes, cellular component and molecular function categories surpassing a DBGGA |zscore| ≥ 2.24 at any one time point for the MAA infected host versus PBS. (XLSX)

S9 Table List of all mechanistic genes (DBGGA |zscore| ≥ 2.24) found for all GO categories for the MAP infected host condition. (XLSX)

S10 Table List of all mechanistic genes (DBGGA |zscore| ≥ 2.24) found for all GO categories for the MAA infected host condition. (XLSX)

S11 Table List of the common and unique mechanistic genes for pathways and GO terms listed in Table 3 comparing MAP and MAA conditions at the early and late stages. (XLSX)

S12 Table Model interrogation results for pathways listed in Table 3. (XLSX)

S13 Table Toll-like Receptor Pathway Bayesian Model Interrogation Results of Gene-to-Gene Relationships and Mechanistic Genes. (A) Interrogation results found in common between the MAP and MAA conditions. (B) Interrogation results found unique to the MAP condition. (C) Interrogation results found unique to the MAA condition.
(XLSX)
