# Supplementary material for: Systems Analysis of Early Host Gene Expression Provides Clues for Transient Mycobacterium avium ssp avium vs. Persistent Mycobacterium avium ssp paratuberculosis Intestinal Infections
Source: PLoS One. 2016 Sep 21;11(9):e0161946. doi: 10.1371/journal.pone.0161946 (PMC5031438; doi:10.1371/journal.pone.0161946)
Supplement: S1 File — (DOCX) [file pone.0161946.s001.docx]

**S1 Materials and Methods**

**1.0 Systems Biology Computational Pipeline.**

Seralogix’s systems biology computational pipeline (SBCP) was developed through support from NIAID, NHGRI and NIFA [[1-5](#_ENREF_1)]. The systems biology computational pipeline (**SBCP**) is capable of managing data importation; complete processing of raw microarray and RNAseq read alignment and mapping to genes, differential gene and pathway and gene ontology (**GO**) analysis/modeling, and delivering results via the web for real-time interactive viewing (S1 Fig). It can process transcriptomic, proteomic, and metabolomics data, such as those collected from NGS, microarray, mass spec and other high throughput platforms. A main SBCP component is the Dynamic Bayesian Gene Group Activation (**DBGGA**) tool which applies Bayesian methods to score gene sets and identify mechanistically influential regulatory genes within pathways and Gene Ontology (**GO**) categories (See section 2.0 below). The SBCP also enables comprehensive cross-comparisons of the “omic” data inputs to support multi-perturbation and time-course studies used to decipher key mechanistic relationships underlying the compared observations. The results are provided to researchers via a web based reporting system.

S1 Fig. Computational pipeline for systems biology embraces a process of iterative refinements as Bayesian models are developed to capture the genetic regulatory mechanism and dysfunctions of diseases.

The SBCP was employed to process microarray data pre- and post MAP and/or MAA host infections.

1. **Variance Smoothing Bayesian Z-scoring for Differential Gene Analysis**

The Z-scores computed in this method are similar to that of Baldi and Long [[6](#_ENREF_6), [7](#_ENREF_7)] and are based on a Bayesian estimator of variance to better deal with low and highly expressed genes. At the simplest level, the method models log-expression values by independent normal distributions, parameterized by corresponding means and variances with hierarchical prior distributions. The method derives point estimates for both parameters and hyperparameters, and regularized expressions for the variance of each gene by combining the empirical variance with a local background variance associated with neighboring genes (variance smoothing). An additional hyperparameter, inversely related to the number of empirical observations, determines the strength of the background variance. Simulations show that these point estimates, combined with a t -test, provide a systematic inference approach that compares favorably with simple t -test or fold methods, and partly compensate for the lack of replication [[7](#_ENREF_7)].

**2.0 Dynamic Bayesian Gene Groups Activation (DBGGA) pathway and gene ontology analysis methods**

DBGAA employs machine learning methods which, owing to their probabilistic nature, has advantages over other computational approaches such as clustering (or naïve correlation) [[8](#_ENREF_8)], Boolean models [[9](#_ENREF_9)], Differential Equation [[10](#_ENREF_10)], Relevance networks [[11](#_ENREF_11)], Graphical Gaussian nets [[12](#_ENREF_12)], Genetic algorithm [[13](#_ENREF_13)], Neural networks [[14](#_ENREF_14)], Static Bayesian networks [[15](#_ENREF_15)] and dynamic models [[16](#_ENREF_16)]. These advantages include the modeling of uncertainty, hidden variables, automated learning, and inference of regulatory network relations. Moreover, Bayesian networks are biologically interpretable and can be scored rigorously against observation data, rendering them preferable to complex non-linear models (e.g. neural nets). A large body of work has successfully applied Bayesian networks in various network learning techniques and for interpretations of biological systems [[15](#_ENREF_15), [17-21](#_ENREF_17)]. The DBGGA applies Dynamic Bayesian networks that supports time-series data of equal or unequal time sampling intervals and enables the modeling of complex temporal processes that may include feedback loops [[22](#_ENREF_22)]. The DBGGA method supports continuous variables and uses all interrelated gene data on each pathway and/or Gene Ontology (GO) group for scoring pathway/GO perturbation. This approach *avoids* information loss associated with discretization methods commonly employed by other pathway analysis programs such as IPA, David, GeneGO in which only previously determined statistically significant genes are used to determine pathway activity. Furthermore, the use of DBN network models enables the extraction of additional knowledge of correlation between networked genes and supports data simulation for “what if” type of analysis.

Through the DBN-based analysis and modeling, DBGGA identifies groups and individual genes that as a whole represent the perturbation in a pathway or biological process (i.e. GO term) over time. DBGGA can determine the differences and commonalities between conditions and disease states and can determine what genes are the significant sources of the perturbation (such genes are designated as “influential regulator genes (IRG)” or sometimes as “mechanistic genes”). DBGGA relies on posterior Bayesian network sampling and interrogation methods to measure a complete pathway perturbation between conditions and can determine a single gene’s influence in a pathway/GO gene set and transforms this influence measure (log-likelihood deviation) to a z-score test statistic. Once trained, DBGGA method can also identify mechanistic gene regulatory relationships that are most influential as regulator genes and hubs in the context of biologically related genes and their interactions. The DBGGA scoring is systematically applied across all known pathways/GO groups providing a comprehensive biological system profile defining overall phenotypic responses by scoring all pathways and GO groups and their associated influential regulator genes (IRG) or mechanistic genes.

The underlying DBGGA computational procedures rely on Dynamic Bayesian Networks. A DBN is a directed, acyclic graphical (DAG) model of a stochastic process. The DBN technology has been extensively tested on numerous analysis projects. The DBN consists of time-slices (or time-steps), with each time-slice containing its own variables (e.g. gene/protein nodes). It is defined as the pair (*BN*_0_, *BN*_→_ ) where *BN*_0_ defines the prior or initial state distribution of the state variables *P*(*Z_t=_*_0_) and *BN*_→_ is a two-slice temporal Bayesian network (2TBN) that defines the transition model *P*(*Z_t_*|*Z_t_*_-1_) as follows:

$$P\left( Z_{t} | Z_{t-1} \right)=\prod_{i=1}^{N} P(Z_{t}^{i}|Pa\left( Z_{t}^{i} \right))$$

Where $Z_{t}^{i}$ is the *i*-th node (gene, protein, etc) at time slice *t*. $Z_{t}^{i}$ can be a hidden node, an observation node, or another type of specialized node for classification. *Pa*($Z_{t}^{i}$) are the parents of $Z_{t}^{i}$ which can be in the same or previous time-slice. The parameters *θ* defining the conditional probability $P(Z_{t}^{i}|Pa(Z_{t}^{i}$ )) associated with each node variable $Z_{t}^{i}$, may then be learned from a training dataset by application of the Expectation-Maximization (EM) algorithm [[23](#_ENREF_23)]. The EM algorithm determines the parameters $\theta_{ML}=\mathrm{argmax}_{\theta}P(Z|\theta)$ maximizing the model probability given the training datasets. The training dataset, *D*, is a set of observations (e.g. gene expression, protein levels, etc.) for each time slice *t*, over a total sampling time period. In this way, an unbounded temporal sequence length of *T* time-slices can be modeled using a finite number of parameters. The DBN is realized by “unrolling” the 2TBN until there are *T* time-slices (S2 Fig). The joint probability distribution of this DBN is defined by:

$$P(Z_{1:T})=\prod_{t=1}^{T} \prod_{i=1}^{N} P(Z_{t}^{i}|Pa\left( Z_{t}^{i} \right))$$

In addition, to avoid model overfitting, regularizations methods (i.e., Tikhonov regularization [[24](#_ENREF_24), [25](#_ENREF_25)]) are applied in the DBN training process, which is a concern in inferring behaviors of thousands of interlinked genes from a small number of data observations.


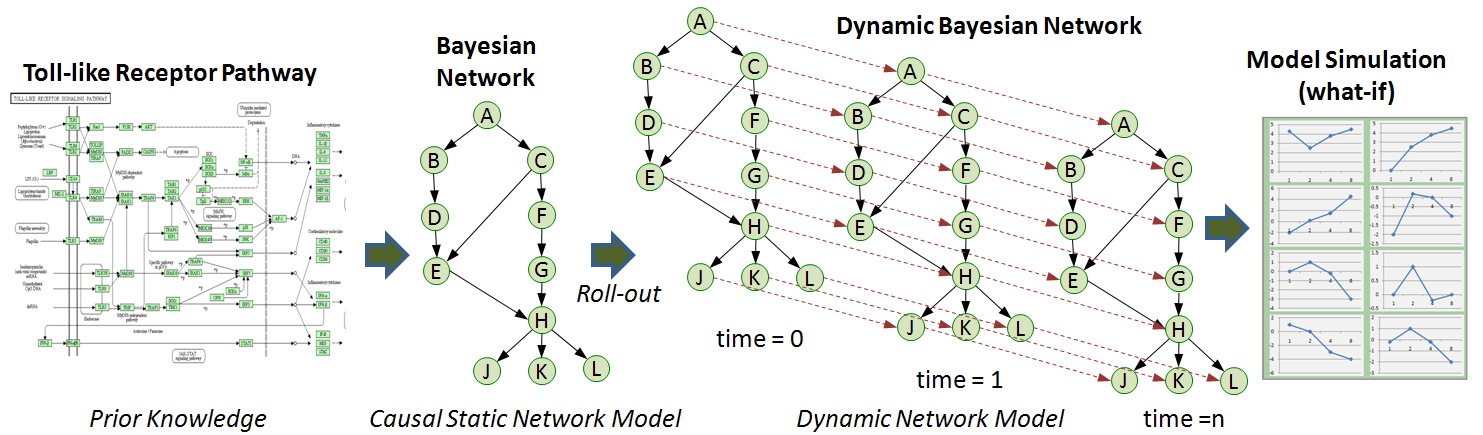


**S2 Fig.** Dynamic Bayesian Network Rollout. Prior network structure knowledge (such as from Kegg) can be used to define the causal network. The causal Bayesian network is rolled out by *n* number of time slices to capture the dynamic relationships.

The DBN model-based comparisons between different experimental conditions are automated to score and select genes or groups of genes that are responsible for the activation/inactivation of pathways, pathway subnets, or biological processes over time as identified by the DBGGA procedure. This scoring procedure essentially relies upon prior network structure knowledge and the ability of Bayesian Network models to assess the statistical likelihood of a given set of data values for the variables (i.e., the network nodes). It needs to be emphasized that this likelihood assesses the entire set of values taken as a whole. This is critical, as the statistical relationships/correlations (i.e., weighted directed network edges) represented by the DAG network structure imply that the expected value (e.g., gene expression level) of a given network node (e.g. a gene) depends on the values of the neighboring parent nodes.

DBGGA scores a given gene or group of genes by evaluating the contribution of the associated data for the gene or group to the Bayesian Network likelihood. This contribution (for a particular time slice, *t*, may be measured by the conditional probability $P(Z_{t}^{i}|\left\{ Z_{t}^{j} | \left( j,t^{'} \right)\neq\left( i,t \right) \right\})$. In practice, the interest is in scoring genes/groups with regard to their contribution in differentiating experiments from controls. Thus, the conditional likelihoods $P(Z_{t}^{i}|\left\{ Z_{t}^{j} | \left( j,t^{'} \right)\neq\left( i,t \right) \right\}$ are computed for both the control-condition and the experimental-condition data with the DBN model parameters optimized (trained) for the control-condition. A two sample, un-paired T-test is then used to assign p-values to the hypothesis that the gene group or an individual gene experimental-condition log-likelihoods are significantly lower than their control-condition log-likelihoods as assessed by this control condition DBN model. The lower log-likelihood implies that the experimental condition for the given gene group is divergent (poorer model fit) from the control condition. These p-values are then converted to z-score equivalents, such that a high z-score indicates a strong contribution to the distinguishing of experiment from control. In some rare cases the experimental data has been observed to have better fitting data to the control model than the control data resulting in a negative z-score. In these cases the z-score is floored to zero since we are only interested in the hypothesis that the experimental data is a worse fit to the control model. Since the log-likelihoods cannot distinguish the direction of perturbation (i.e., up-regulated vs. down-regulated in the case of an individual gene or induced versus suppressed for pathways and GO groups), the last step for computing the final score is to multiply the Bayesian z-scores by +1 if the average of the experimental condition data is greater than the control condition data and -1 if it is lower. This transformation from likelihood to a z-score is referred to hereafter as the **DBGGA Bayesian Z-score (Bayesian z-score)**. An individual gene that meets a user specified significance threshold is termed an **influential regulator** **gene (i.e., mechanistic)**, because this gene contributes significantly to the perturbed state of the pathway in which it is associated.

The DBGGA scoring can be applied at the pathway level which is a group of interconnected genes. The data for all the genes in the pathway from the perturbed experimental condition is scored against the control DBN model as described above. This results in the ability to compare the conditional probability-based score with those of several hundred other pathways that were scored as part of the system’s analysis. Thus, it is possible to rank and select the pathways and/or GO groups that are most perturbed under differing experimental/disease conditions. Similarly, individual genes may be scored within the pathway models. Fig 3 in main manuscript illustrates the comparison of pathway scores (as a heatmap) for the set of pathways that compares *Mycobacterium avium ssp. paratuberculosis* (MAP) condition and *Mycobacterium avium ssp. avium* (MAA). condition as they change over time. Fig 5 in the main manuscript are the network representation of the Tight junction pathway comparing MAP and the MAA conditions at 30 minutes post infection.

For each experimental condition, the trained network model can be interrogated for IRGs/mechanistic genes (meets a Bayesian z-score threshold defined by the user) across all time points and the strength (correlation) of connections between interconnecting genes. Each pathway model can produce a heat map of candidate mechanistic gene scores (at any selected threshold) by time point.

*2.1 Validation of DBGGA Pathway/GO Perturbation Determination*. Statistical methods employing synthesized data and real gene expression data were employed to determine sensitivity, selectivity and robustness of DBGGA algorithms to detect pathway/GO gene group and individual gene perturbations within the context of the pathway/GO interactions (network). The DBGGA method was found to outperform traditional methods such as hypergeometric-based enrichment detection techniques (e.g. GeneMerge [[26](#_ENREF_26)] and GOminer [[27](#_ENREF_27)]) in determining subtle changes in gene expression levels for groups of interrelated genes. Sensitivity and selectivity for distinguishing perturbed genes while increasing additive white noise also proved more robust than these traditional methods. Note, the identification of significant gene perturbation by DBGGA has been experimentally confirmed by qRT-PCR in several recent studies [[28-33](#_ENREF_28)]. Temporal data was synthesized representing control (healthy state) for use as training and testing. Sensitivity testing cases representing the treatment/experimental state were derived from actual gene expression data by perturbing expression levels of a selected number of genes (induced known spiked data). From this data, validation tests were conducted for: 1) the Dynamic Bayesian Gene Group Activation (DBGGA) methods applicable to both pathways and Gene Ontology functional categories, and 2) discriminating which genes contribute significantly to overall pathway/GO perturbation (i.e., mechanistic genes).

The DBGGA method was compared to the statistical-based hypergeometric model under conditions injecting varying and controlled white noise upon the testing data. The hypergeometric model computes the discrete probability of selecting *r* number of changed genes from *k* changed genes on the microarray or RNAseq expressions, with a total population of size *n* on the microarray, and is used in many currently available tools including GeneMerge [[34](#_ENREF_34)] and GOminer [[35](#_ENREF_35)] and others. This example bench-marked a DBGGA model for identifying gene group activation from the TLR6->TNF subnet model of Toll-like Receptor Pathway. The TLR6-TNF subnet contains a reaction chain comprised of 37 genes. Multiple temporal datasets were generated; each comprised of 100 experiment samples (representing an infection host response) and 200 healthy samples, the later was randomly split into equal sets of training and (blind) test data. Each sample represented 9950 genes measured over four time points (0, 12, 24, 48 hrs). Gaussian sampling around the empirical gene expression means and multiplicative factors [1x-4x], of the standard deviations were used to generated the data sets representing varying levels of white noise. Model training was conducted with control data noised equivalent to the empirical data (1x noise). To represent the experimental/diseased state for this example, a set of 12 genes were perturbed by a 1.5 fold up regulation - a perturbation that under classical, individual fold analysis would not be considered significantly “changed”. This model was designed to demonstrate the sensitivity of DBGGA over traditional methodologies when dealing with groups of genes with subtle changes, i.e. guard against Type I errors (False Negatives).

To determine gene activation thresholds - required by the hypergeometric method - a classical p=value test at a significance level of 0.05 ($\alpha=0.05$) was employed. Classification rates for both models were then measured in the presence of increasing levels of white noise. S3 Fig shows the classification rates (y axis) for DBGGA (red) and hypergeometric (blue) versus Noise Multiplication Factor (x axis). It is observed that the DBGGA is more sensitive for correctly classifying the known perturbed genes as positive or “experimental/diseased case”, while the hypergeometric was essentially ineffective in correct classification of these positives. As noise increased, it was observed that the DBGGA method retained its sensitivity, while specificity of both models dropped significantly.

To evaluate the sensitivity of the DBGGA with respect to the number of changed genes within a single pathway, the correct classification rate of DBGGA versus hypergeometric at 3,7,14, and 28 changed genes were examined. Perturbations ranging from 1.0 to 3.0 fold change above empirical mean control values were generated. As described above, this analysis employed the same TLR6-TNF subnet. The results are shown in S4 Fig. Again, it was observed that the DBGGA method performed very well in the lower expression levels and that correct classification rate remains robust even as the number of perturbed genes on the subnet is reduced from 28 to 3. The hypergeometric method performs best when a large number of genes are perturbed, but degrades quickly as the number of perturbed genes is reduced to three in which case the hypergeometric method detected no true subnet activations whereas the DBGGA method was able to reach high correct positive classification rates with as little as three perturbed genes at 2-fold change.


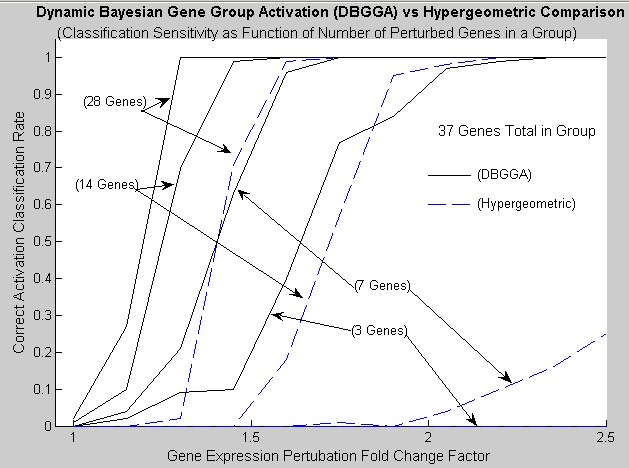


**S4 Fig**. Comparison of DBGGA vs. hypergeometric for correct activation classification as a function of the amount of perturbation and number of perturbed genes within a group.


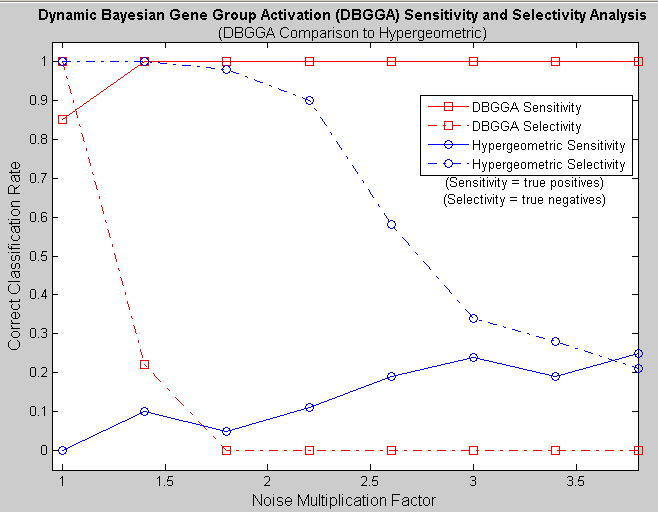


**S3 Fig**. DBGGA sensitivity and specificity is compared to the hypergeometric method for determining gene group over-expression. The DBGGA method is superior over the hypergeometric method especially for lower expression value situations.

*2.2 Validation of Mechanistic Gene Determination.* Mechanistic genes are those genes found to influence the perturbation of a set of interconnected genes in a pathway or GO group. DBGGA produces a different measurement than simply finding a significantly perturbed gene in isolation from the surrounding parent and child network genes as described previously above for the DBGGA methods. Although the DBGGA mechanistic gene determination method measures how much influence the gene has in the overall pathway/GO perturbation, this scoring approach does correlate well with genes scored by the traditional statistical fold or p-value/z-score techniques.

A total of five datasets were used for this statistical tests/validation. Data sets were obtained from multiple sources having varying time series’, gene sets, array manufactures, and experimental conditions. The data sets used included:

| **Id** | **Dataset Name/Condition** | **Array Organism** | **Time Series** |
| --- | --- | --- | --- |
| **A** | B melitensis expression in HeLa cell culture | B melitensis | 4, 12 hrs |
| **B** | Mycobacterium avium ssp. Avium in bovine | bovine | 30,60,120,240,480,720 min. |
| **C** | In vivo TPR-deficient NeuralTube in mouse embryo | mouse | 9,10 days |
| **D** | Salmonella (ZA21) mutant challenged bovine | bovine | 15,30,60,120,240,480,720 min. |
| **E** | Folate Acid Dose Response 2000µM vs 1.7 µM in cancer cells | human | 1 timepoint |

Datasets were processed using the standard Seralogix pipeline, and then analyzed using traditional statistical tools to compute fold change and z-score and DBGGA analysis for both pathway and GO-based models and mechanistic gene identification. The results from the fold and z-score analysis were used as “truth” data for comparison to the model-based results for computing sensitivity (true positive) and specificity (true negative), false positives rates and false negative rates results.

False positive rate testing was done comparing data in the control or healthy state to portions of the same control data for all tests. Traditional tests were executed in a “leave one out” manner for all repetitions, while pathway and GO models were trained to reflect the organism system in healthy state and compared, one experimental sample repetition at a time.

| **Gene Perturbation False Positive Rate Testing Results** | | | | |  |
| --- | --- | --- | --- | --- | --- |
| **Data Set** | | **2-Fold Change** | **ZScore (95% conf.)** | **DBGGA Pathway Model** | **DBGGA GO Model** |
| A | | 0.347% | 2.662% | 0.826% | N/A |
| B | | 3.013% | 7.262% | 0.000% | 0.184% |
| C | | 0.319% | 0.428% | 0.038% | 0.000% |
| D | | 2.196% | 4.742% | 0.032% | 0.001% |
| E | | 0.762% | 0.078% | 0.005% | 0.001% |

Gene modulation truth data was obtained for False Negative Rate testing by considering all genes that surpassed a 2-fold test and 95% confidence on zscore statistical testing as truly changed genes. The pathway and GO models were the same as previously trained in false positive rate testing and presented repetitions of experimental data.

| **Gene Perturbation False Negative Rate Testing Results** | | | | |
| --- | --- | --- | --- | --- |
| **Data Set** | **2-Fold** | **ZScore** | **Pathway Model** | **GO Model** |
| **A** | 0.826% | 0.826% | 0.826% | N/A |
| **B** | 0.065% | 0.032% | 0.032% | 0.000% |
| **C** | 4.378% | 2.504% | 1.767% | 2.749% |
| **D** | 19.908% | 2.298% | 0.226% | 0.497% |
| **E** | 0.001% | 0.428% | 4.015% | 6.252% |

Both pathway and GO naïve model architectures performed markedly better on specificity (true negative) testing than traditional 2-fold and 95% confidence zscore testing, indicating a better tolerance for noise and artifacts. We had expected to see the pathway models perform somewhat better on specificity than the naïve GO-based models. However, the GO models performed better by a statistically insignificant margin.

| **Gene Perturbation Specificity and Sensitivity Testing Results** | | | | |
| --- | --- | --- | --- | --- |
| **Model/Analysis** | **2-Fold** | **ZScore** | **Pathway Model** | **GO Model** |
| Specificity (true neg. rate) | 0.986 | 0.969 | 0.998 | 0.999 |
| Sensitivity (true pos. rate) | 0.949 | 0.987 | 0.986 | 0.976 |

Sensitivity testing shows that the zscore, pathway and GO models are statistically a dead heat, with 2-Fold performing significantly less well. It is important to note that the true sensitivity levels can be affected by the method in determining “truth data”.

1. Drake, K. and (PI), *BWA Host-Pathogen Innate Immune S/W Analysis Tool, Grant # 2R44AI58362-03A1 SBIR Phase II* National Institute of Allergies and Infectious Diseases.

2. Drake, K. and (PI), *Bioinformatics for Immune Response Biosignature Analysis, 2R44AI055061-02 SBIR Phase II* National Institute of Allergies and Infectious Diseases.

3. Drake, K. and (PI), *Host-Pathogen Interaction Network Learning from In Vivo Gene Co-Expression, 1R43AI084223-01 SBIR Phase I.* National Institute of Allergies and Infectious Diseases.

4. Drake, K. and (PI), *Computational Methods for Functional Genomic Discovery from Gene Knockout, SBIR Phase II 2R44HG004457-02, National Human Genome Research Institute*.

5. Zhou, H. and K. Drake, *Systems Biology Analysis and Modeling of Complex "OMIC" Data: A Service Center Approach.* Award Number 2012-67015-31217 (Prime Agreement) from US Department of Agriculture (USDA), National Institute of Food and Agriculture (NIFA) (Prime Sponsor) (CFDA 10.310). .

6. Baldi, P. and G.W. Hatfield, *DNA Microarrays and Gene Expression: From Experiments to DataAnalysis and Modeling*. 1st ed. 2002: Cambridge University Press.

7. Baldi, P. and A.D. Long, *A Bayesian framework for the analysis of microarray expression data: regularized t -test and statistical inferences of gene changes.* Bioinformatics, 2001. **17**(6): p. 509-19.

8. Eisen, M.B., et al., *Cluster analysis and display of genome-wide expression patterns.* Proc. Natl. Acad. Sci Genetics, 1998. **95**: p. 14863-14868.

9. Bornholdt, S., *Boolean network models of cellular regulation: prospects and limitations.* J. R. Soc. Interf., 2008. **5**: p. S85–S94.

10. Chen, T., H.L. He, and G.M. Church. *Modeling gene expression with differential equations*. in *PacSympo. Biocomput.* 1999.

11. Butte, A. and I. Kohane. *Mutual information relevance networks: functionalgenomic clustering using pairwise entropy measurements*. in *Proceeding of the Pacific Symposium on Biocomputing*. 2000.

12. Opgen-Rhein, R. and K. Strimmer, *From correlation to causation networks: a simple approximate learning algorithm and its application to high-dimensional plant gene expression data.* BMC Systems Biology, 2007. **1**(37).

13. Sakamoto, E. and H. Iba. *Inferring a system of differential equations for a gene regulatory network by using genetic programming*. in *Proceedings of the IEEE Congress on Evolutionary Computation*. 2001.

14. Chen, C.F., X. Feng, and J. Szeto, *Identification of critical genes in microarray experiments by a Neuro-Fuzzy approach.* Comput Biol Chem. , 2006. **30**(5): p. 372-81.

15. Friedman, N., et al., *Using Bayesian networks to analyze expression data.* J. Comput. Biol, 2000. **7**: p. 601–620.

16. Hecker, M., et al., *Gene regulatory network inference: Data integration in dynamic models—A Review.* BioSystems, 2009. **98**: p. 86–103.

17. Sachs, K., et al., *Causal protein-signaling networks derived from multiparameter single-cell data.* Science, 2005. **308**: p. 523–529.

18. Troyanskaya, O.G., et al., *A Bayesian framework for combining heterogeneous data sources for gene function prediction (in Saccharomyces Cerevisiae).* Proc Natl Acad Sci U S A, 2003. **100**: p. 8348–8353.

19. Beaumont, M.A. and B. Rannala, *The Bayesian revolution in genetics.* Nat Rev Genet, 2004. **5**: p. 251–261.

20. Heckerman, D., *A tutorial on learning with Bayesian networks*, in *In: Jordan MI, Learning in graphical models*. 1998, in Jordan MI, Dordrecht: Kluwer Academic. p. 301–354.

21. Imoto, S., et al., *Combining microarrays and biological knowledge for estimating gene networks via Bayesian networks.* J Bioinform Comput Biol., 2004: p. 77-98.

22. Needham, C.J., et al., *A Primer on Learning in Bayesian Networks for Computational Biology.* PLoS Comput Biol, 2007. **3**(8).

23. Lauritzen, S.L., *The EM algorithm for graphical association models with missing data.* Computational Statistics and Data Analysis, 1995. **19**: p. 191–201.

24. Tikhonov, A.N. and V.Y. Arsenin, *Solutions of ill posed problems* 1977, Washington, D.C.: Winston.

25. Hoerl, A.E. and R.W. Kennard, *Ridge Regression: Biased Estimation for Nonorthogonal Problems.* Technometrics, 1970. **42**(1): p. 80-86.

26. Castillo-Davis, C.I. and D.L. Hartl, *GeneMerge-post-genomic analysis, data, and hypothesis testing.* Bioinformatics, 2003. **19**: p. 891-892.

27. B. Zeeberg, B., et al., *GoMiner: A Resource for Biological Interpretation of Genomic and Proteomic Data.* Genome Biology, 2003. **4**(4).

28. Adams, L.G., et al., *Multi-comparative systems biology analysis reveals time-course biosignatures of in vivo bovine pathway responses to B.melitensis, S.enterica Typhimurium and M.avium paratuberculosis.* BMC Proc, 2011. **5 Suppl 4**: p. S6.

29. Khare, S., et al., *Systems Biology Analysis of Gene Expression during In Vivo Mycobacterium avium paratuberculosis Enteric Colonization Reveals Role for Immune Tolerance.* PLoS One, 2012. **7**(8).

30. Lawhon SD, et al. *Role of SPI-1 Secreted Effectors in Acute Bovine Response to Salmonella enterica Serovar Typhimurium: A Systems Biology Analysis Approach*. PLoS ONE, 2011. **6**, e26869 DOI: 10.1371.

31. Rossetti, C.A., et al., *Transcriptional profile of the intracellular pathogen Brucella melitensis following HeLa cells invasion.* Microbial Pathogenesis, 2011. **51**: p. 338-344.

32. Rossetti, C.A., et al., *Systems biology analysis of Brucella infected Peyer's patch reveals rapid invasion with modest transient perturbations of the host transcriptome.* PLoS One, 2013. **8**(12): p. e81719.

33. Weeks, J.N., et al., *Brucella melitensis VjbR and C12-HSL regulons: contributions of the N-dodecanoyl homoserine lactone signaling molecule and LuxR homologue VjbR to gene expression.* BMC Microbiol, 2010. **10**: p. 167.

34. Castillo-Davis, C.I., and Hartl, Daniel L, *GeneMerge-post-genomic analysis, datamining, and hypothesis testing.* Bioinformatics, 2003. **Vol. 19** (no. 7): p. pages 891-892.

35. Zeeberg, B., Feng, Weimin , Wang,Geoffrey , Wang, May, Fojo, Anthony ,Sunshine, Margot ,Narasimhan, Sudarshan ,Kane, David , Reinhold, William , Lababidi, Samir , Bussey, Kimberly , Riss, Joseph, Barrett,Carl , and Weinstein, John *GoMiner: A Resource for Biological Interpretation of Genomic and Proteomic Data. 2003 4(4):R28 (published )* Genome Biology, 2003.
