# Supplementary material for: Systems Analysis of Early Host Gene Expression Provides Clues for Transient Mycobacterium avium ssp avium vs. Persistent Mycobacterium avium ssp paratuberculosis Intestinal Infections
Source: PLoS One. 2016 Sep 21;11(9):e0161946. doi: 10.1371/journal.pone.0161946 (PMC5031438; doi:10.1371/journal.pone.0161946)
Supplement: S2 File — (DOCX) [file pone.0161946.s002.docx]

**S2 Results and Discussion**

**Discussion of Pathway and GO Category Differences between MAP and MAA Host Response**

This supplement focuses on the pathways and GO categories that have either: 1) a reversed state of activation between MAP and MAA host response; or 2) has unique pathway/GO perturbations in comparison to the other infectious condition. Such behavior could be related to the differences in the virulent factors of the MAP infected host versus the MAA infected host and the differences in the effectiveness of the host immune response resulting in the persistent or transient infection. To aid in our analysis, we define an early stage (30 and 60 minutes post infection) and late stage (480 and 720 minutes post infection) host immune response time frames to determine which gene relationships may be related to the pathogen invasion (early stage) and which may be associated to an effective or ineffective host defensive response during the late stage. From an immune response perspective, the biological processes found to have a reversed state of activation and/or repression are listed in Table 3(A) (see main text) for pathways and GO immune categories and separated by early and late stages. The pathways and GO categories found to be unique to the MAP condition or the MAA condition are listed in Table 3(B) and (C) respectively, also separated into early and late stages. Here we have focused on the pathways and GO categories that are discriminatory between MAP and MAA infected tissues.

**MAP influence on the mucosal barrier and cell adhesion processes**

There is an interesting pattern that emerges from the Table 3(A) and (B) that suggests that MAP manipulates the cell adhesion processes of the host as evident by the early stage reversed states of perturbation of the Gap Junction, Adherens Junction, and Focal Adhesion junction pathways as well as the MAP condition’s unique repressed state of the Tight Junction pathway and the GO term “cell-cell adhesion mediated by integrin”. This suggests that MAP host invasion may be modulating critical cell adhesion processes in a complex manner. The junction and cell adhesion related mechanistic gene DBGGA z-scores, are shown in the MAP-MAA comparative heatmap of Fig 6A (see main text). For the MAP condition at 30 or 60 minute post infection, it is apparent that, in general, the magnitude of perturbation is greater and has more down regulated genes for MAP than for the MAA condition.

Prior studies have shown that up regulation (increased gene expressions) of these junction/adhesion pathways may lead to strengthening the intestinal mucosal barrier while down regulation (disrupted gene expression) may result in weakening of the this immune barrier. Reported previously [[12](#_ENREF_12)], the MAP infection caused a marked decrease in the Trans-Epithelial Resistance (TER) of an *in vitro* model polarized epithelial cells, suggesting that increased permeability of *in vivo* host intestinal epithelium may facilitate bacterial invasion through the intestinal epithelium. The mucosal barrier has three major components, the mucus layer, the epithelial glycocalyx and the surface epithelium itself, whose integrity largely depends on Tight Junction function [[20](#_ENREF_20)]. The Tight Junction would appear to play a major role in the pathogenic difference between the MAP and MAA host response. To more fully understand these differences, we interrogated the Tight Junction Bayesian network model parameters, specifically; we considered the mechanistic genes and associated gene-to-gene correlation weights that were learned from the host response data for the MAP and MAA treatment and control conditions. In this analysis, the Tight Junction pathway was found to have several significant mechanistic genes and gene-to-gene correlation differences as graphically illustrated in the network models of Fig 5A and B (main text). These network models are comprised of 72 gene nodes and 234 connecting arcs. In these networks, the orange encircled gene nodes indicate mechanistic genes for that time point illustrates just how different the host response is between treatment conditions during the early stage (30 minutes post infection) and the late stage (720 minutes post infection). The network arcs connect a parent gene node to a child gene node where the arc width indicates increasing absolute value correlation weights and their color indicate the direction of correlation (refer to Fig 5 legend). To identify these relationships, we developed an algorithm to perform a comparative analysis between models to identify common (Table 4(A) and significantly divergent network normalized correlation weights and mechanistic genes (Table 4(B) and (C)). To create Table 4(A) the absolute value of normalized arc weights (norm. arc wt.) for both MAP and MAA models had to be greater than 0.2 (i.e., high dependency probability between connecting gene nodes) and either the parent gene or child gene had to have a mechanistic gene |DBGGA z-score| ≥ 1.96. Table 4(B) was created based on the criteria that only the MAP condition had |norm. arc wt.| ≥ 0.2, and absolute value difference between MAP and MAA norm. arc wt > 0.1, and either the MAP parent gene or child gene had to have a mechanistic gene with |DBGGA z-score| ≥ 1.96. Table 4(C) was created based on the same criteria as Table 4(B) except applied to the MAA host response model. The interesting observations in Table 4(B) are the 13 unique early stage (30-60 min. post infection) relationships identified for the MAP condition in comparison to only 5 early stage relationships for MAA (Table 4(C)). This suggests that the MAP Tight Junction pathway time-course response data resulted in a larger percentage of higher probability gene-to-gene relationships than MAA and that MAP had more dominate early stage pathway modulation. Interestingly, the unique MAP relationships (Table 4(B)) had only three late stage identified relationships while for MAA there was no late stage unique relationship. Referring again to Table 4(A), there were ten common MAP-MAA early stage relationship pairs each having similar correlative weights and direction, but even though the correlations are similar, the magnitude, direction, and timing of the first time point mechanistic gene scores are quite dissimilar. It is assumed that relationships that include strongly down regulated gene members and reversed correlative weights between conditions may imply points of disruption (i.e., phenotypic mechanisms) in the Tight Junction pathway. For example, from Table 4(A) and (B) the most striking candidate mechanistic relationships are CLDN7→TJP1, CLDN7→TJP2, CLDN7→TJP3, CDK4→CSDA, ACTB→MYL2, ACTG1→MYL2, ACTN1→MAGI3, CLDN7→MPDZ, CLDN7→INADL, JAM3→TJP1, and ACTN3→MAGI3. The strongly down regulated CLDN7 (claudin 7) gene encodes a member of the claudin family which includes integral membrane proteins and components of tight junction strands that serve as a physical barrier to prevent solutes and water from passing freely through the paracellular space between epithelial or endothelial cell sheets and also play critical roles in maintaining cell polarity and signal transductions. CLDN7 interacts with five other genes having no significant levels of expression which suggest that the down regulation of CLDN7 may disrupt down-stream expressions of the associated genes: TPJ1, TJP2, TJP3, MPDZ, and INADL. The genes TJP1, 2, and 3 encodes tight junction proteins that are members of the membrane-associated guanylate kinase homolog family. This protein functions as a component of the tight junction barrier in epithelial and endothelial cells and is necessary for proper assembly of tight junctions. The gene MPDZ encodes a protein of the N-methyl-D-aspartate receptor (NMDAR) signaling complex that may play a role in control of α-amino-3-hydroxy-5-methyl-4-isoxazolepropionic acid receptor (AMPAR) potentiation and synaptic plasticity in excitatory synapses. The gene INADL encodes a protein with multiple PDZ domains. PDZ domains mediate protein-protein interactions, and proteins with multiple PDZ domains often organize multimeric complexes at the plasma membrane. This protein localizes to tight junctions and to the apical membrane of epithelial cells. Prior research showed that CLDN7 deficient mice severely disrupted the integrity of the mucosal/epithelial barrier [[21](#_ENREF_21)]. The gene JAM3 (junction adhesion molecule C) encodes a protein that is a member of the immunoglobulin superfamily that is localized in the tight junctions between high endothelial cells. JAM3 is a widely expressed adhesion molecule regulating cell adhesion, cell polarity and inflammation. ACTN3 (actinin, alpha 3) encodes a member of the alpha-actin binding protein gene family. Interestingly, in the late stage, the Tight Junction pathway for the MAP condition becomes strongly activated. In contrast, the MAA early stage Tight Junction pathway host response was not significantly perturbed, suggesting that MAA may not be as efficient as MAP to cross the epithelial barrier. Interestingly, by the late stage, the MAA condition Tight Junction perturbation did become significantly repressed (see previous Table 3(A) late stage). Note that we conducted model interrogations on all the pathways listed in Table 3 and results provided in Supplemental Table S12.

Along with the Tight Junction, the Gene Ontology (GO) term “cell-cell adhesion mediated by integrin” was also significantly repressed at the early stage for the MAP condition. Cell adhesion serves to facilitate trafficking and migration of T lymphocytes into sites of inflammation, movement of lymphocytes within the rich environment found in extravascular tissue, and the physical interaction between antigen-reactive T cells and antigen-presenting cells that is required for efficient T-cell activation [[22](#_ENREF_22)]. The repressed junction/adhesion related pathways and their associated genes suggest that MAP may disrupt T lymphocyte recruitment that helps explain the lack of chronic inflammation observed in the MAP infected ileal loops and subvert mucosal healing [[12](#_ENREF_12)]. It has been proposed by others [[23](#_ENREF_23)] that some bacteria survival mechanism in mucosal epithelial cells is for the bacteria to hijack integrin-linked kinase to stabilize focal adhesions and block cell detachment of infected cells. The rapid turnover and exfoliation of mucosal epithelial cells provides an innate defense system against bacterial infection. The significant repression and reversed state of the GO category, “epithelial cell proliferation”, (Table 3(A) early stage) provides evidence of MAP interference with this important host defensive process and suggests that bacteria such as MAP may be able to subvert this immune defense mechanism and colonize the epithelium more efficiently and survive. The repressed state of the GO term “epithelial cell proliferation” (mechanistic gene scores shown in Fig 5(B)) is dominated by the down regulation of FGFR2, BAX, WDR77, CTNNB1 and BCL2L2. FGFR2 (Fibroblast Growth Factor Receptor 2) encodes a protein that is a member of the fibroblast growth. Furthermore, as noted early for the reversed activation state of the GO category “epithelial cell proliferation”, the MAA host response is strongly activated, suggesting a strengthening of the mucosal/epithelial barrier and decreasing the efficiency of MAA invasion.

**Calcium signaling modulation by MAP and MAA**

The Calcium signaling (CS) pathway was strongly activated in the early stage for MAP infected host but was close to being significantly repressed in MAA (Table 3(A)), suggesting MAP infection has influence on this process during invasion and possibly related to MAP persistence. Calcium signaling plays an important role in a broad range of regulatory effects on enzymes and proteins and influence on other major pathways including MAPK Signaling, Apoptosis, Long-term Potentiation, Long-term Depression, Phosphatidylinositol Signaling and others. From the CS Bayesian models, it was found that the main dissimilar gene-to-gene relationships between MAP and MAA were CALM→NOS2, CAMK2D→CALM, CAMK2A→CALM1, and CAMK2D→CALML5. The genes CAMK2A/D (calcium/calmodulin-dependent protein kinase II, alpha/delta) encode proteins involved in calcium transport and also participates in type II interferon signaling and epidermal growth. CALM is strongly down regulated in the MAP infected host but up regulated in MAA. CALM1 is not expressed in MAP but highly up regulated in MAA. It has been shown earlier that mycobacterial toxin may inhibit Ca signaling which may lead to decreased phagosome-lysosome fusion and results in increased survival within human macrophages [[24](#_ENREF_24)]. Thus, MAP infection remains persistent in comparison to the host response to MAA.

**Differences in cell mediated immune responses**

By examining the MAA infected host response (Table 3), it appears that the host is mounting a stronger cell mediated immune response as evident by the significant activations of the B cell receptor signaling pathway and the GO categories “Regulation of T-helper 1 type immune response”, “Regulation of adaptive immune response”, “Cytokine production involved in immune response”, “Regulation of innate immune response”, “T cell costimulation”, and “Interleukine-12 (IL12) production. The overall number of unique pathway and GO category perturbations are far less in the MAA condition which supports the observation that MAA is not as pathogenic as MAP. Interestingly, there are a large number of strongly perturbed pathways and GO categories that show significant reversal of activation states between MAA and MAP. For example, at the late stage, the MAA condition has 16 pathways and GO categories that became strongly repressed in comparison to the activated state for MAP (Table 3(A)), suggesting that the host may be struggling to stop the proliferation of MAP while for MAA, the immune response may be shutting down as the host clears the MAA bacteria.

**Differences in Toll-like receptor**

Central to immune defense against microbial pathogens are pattern recognition receptors including the Toll-like receptors (TLRs) which are capable of recognizing conserved microbial patterns including components of the mycobacterium cell wall and nucleic acids. TLRs mediate the activation of cells of the innate immune systems leading to dynamic functions including direct anti-microbial activity, induction of cytokine secretion, triggering dendritic cell maturation, and triggering apoptosis. TLR activation is also capable of modulating the adaptive immune response with a bias towards a Th1 T-cell response. However, the activation of TLRs by mycobacteria may also provide a means of immune evasion and hence, the modulation of TLR activation can influence the ability to properly destroy invading pathogens. In mycobacterial infections, the innate immune system utilizes many pattern recognition receptors (PRRs) to help recognize, phagocytose, and trigger defense mechanisms against invading bacterium. These include mannose receptor (MRC1, MRC2), complement receptors (CR1/2/3/4), and TLRs. Studies in mice have shown that mycobacterial components are engaged by TLR2 in association with TLR1/TLR6, TLR4, or TLR9 (which recognizes mycobacterial DNA) [[25](#_ENREF_25)]. By interrogating our Toll-like receptor pathway models, it is observed that the TLR perturbations are very dissimilar; it was strongly repressed in the late stage for MAP and activated in the MAA condition. Through our Bayesian model interrogation methods, the “Toll-like receptor signaling” pathway was found to have several significant mechanistic genes and gene-to-gene correlation differences and commonalities (Supplemental Table S13 A, B, and C). The common (i.e. similar) relationships include MAP3K8→MAP2K2, MAP3K8→NFKB1, MAP3K8→MAP2K1, and FOS→IL12B. Several interesting early stage dissimilar relationship include TLR9→MYD88, STAT1→CXCL9, STAT1→CXCL10, STAT1→CXCL11, JUN→IL12B, JUN→IL8, JUN→TNF, JUN→IL1B and TLR3→TICAM1 while for the late stage, the relationships include MAP2K7→MAPK8, and MYD88→FADD and the individual mechanistic genes MAP2K2 and IRF7. MAA has a strong early stage up regulated TLR9 while for MAP the early stage response of TLR9 was insignificant. In contrast, for the MAP condition, TLR3 was significantly up regulated early stage only, while TLR9 became significantly down regulated late stage only. Interestingly, TLR2, TLR4, TLR5 and TLR6 were not significantly expressed in either condition, but the gene CD14, which acts as a co-receptor with TLR4 for the detection of bacterial lipopolysaccharide (LPS) was significantly expressed in both MAP and MAA infected hosts (early stage) and become insignificantly expressed late stage. Numerous mycobacterial studies have revealed that TLR2 is involved in the innate recognition and responses in the innate immune cells [[26](#_ENREF_26)], which appears to be contradictory to that found in this *in vivo* study in which TLR3 and TLR9 were the only significantly up regulated TLRs for MAP and MAA respectively. Furthermore, it was found that the mannose receptor, MRC1, and the complement receptor, CR2, were only up regulated in the MAA infected host. It has been reported that rapidly growing, virulent strains of mycobacteria did not activate TLR2 or TLR4 in a transfection system, suggesting it may activate other TLRs [[27](#_ENREF_27)].

TLR3 plays a fundamental role in pathogen recognition and should mediate the production of proinflammatory cytokines necessary for the development of an effective immune response. TLR3 activation should be inducing the activation of IRF3, which in turn, should induce the production of type I interferons (IFNs). However, IRF3 was significantly down regulated in both MAP and MAA conditions in the early stage and there was insignificant expression of any type I IFNs or for type II IFNs. Moreover, the GO categories for Regulation of interferon-beta (IFN-𝛽) and Interferon-gamma (IFN-γ)-mediated signaling were significantly activated in the MAP, but were both repressed in the MAA condition. Interestingly, in the MAP condition (early and late stage), the chemokine, CXCL9, is up regulated, but down regulated in MAA condition (early stage). CXCL9 is known to be a T-cell chemoattractant that is normally induced by IFN-γ. However, the MAP infected host did have several up regulated cytokines that included IL1B, IL4, CCL25, CX3CL1, CCL20, IL7, IL18, CXCL9, and IL10 primarily all during the late stage. IL1B is produced by activated macrophages and is an important mediator of the inflammatory response. IL4 is produced by CD4 T cells specialized in providing help to B cells to proliferate. IL10 can inhibit the synthesis of a number of cytokines including IFNγ, IL2, IL3, TNF and GM-CSF produced by activated macrophages and by helper T cells.

TLR9 (up regulated in MAA only) is preferentially expressed in immune cell rich tissues from cells such as B lymphocytes, monocytes, natural killer (NK) cells, and plasmacytoid dendritic cells. TLR9 signals should lead to the activation of cells initiating pro-inflammatory reactions in the production of cytokines such as type-I interferon and IL-12. Interestingly, IFN-𝛽 was insignificantly expressed in the MAA infected host, but the GO category “Regulation of cytokine production involved in immune response” was significantly activated suggesting MAA was activating some cytokine response. However, the only cytokines found significantly up regulated in the MAA infected host was CCL5, IL18, IL5, TNF, IL12β, IL4, and CXCL9. TNF (tumor necrosis factor) is associated with apoptosis. In both the MAP and MAA infected host, there was activation of apoptosis only in the late stage. CCL5 plays an active role in recruiting leukocytes into inflammatory sites. IL5 stimulates B cell growth and increases immunoglobulin secretion. IL18 together with IL-12 induces cell-mediated immunity following infection with microbial products like LPS.

**Differences in phagocytosis**

Mycobacteria is usually internalized into macrophages via phagocytosis and resides in phagosomes to help escape destruction reportedly by inhibiting phagosome/lysosome maturation. For MAP infected host, the DBGGA analysis found the GO category, “Phagocytosis” biological process, to be activated briefly at 240 minutes p.i, and became significantly repressed in the late stage, while in the MAA infected host it remained strongly activated. Examining the mechanistic genes of “Phagocytosis”, the main differences is the significant up regulation of HMGB1, CORO1A, and CSK for the MAA infected host, early stage, while in the late stage the dominate genes that induced the repressed state of “Phagocytosis” for MAP condition were PIP5K1C, HMGB1, and CORO1A. The gene PIP5K1C encodes the protein, phosphatidylinositol 4-phosphate 5-kinase type-1 gamma involved in a number of cellular functions, but most relevant is that PIP5K1A is required for phagocytosis in the regulation of actin remodeling. The role of actin cytoskeleton remodeling during particle internalization is well established, but the role during the later stages of phagosome maturation (phagosome/lysosome fusion) remains largely unknown. The disruption of this gene may be involved in the MAP pathogenicity by impeding phago-lysosomal fusion and hence, inhibiting phagosome maturation. Further, the repressed state of the “phosphatidylinostial signaling system” in the MAP condition (early stage) provides additional evidence that MAP may disrupt this process. HMGB1 gene encodes the protein, high-mobility group protein B1, which supports the transcription of many genes in interactions with many transcription factors. HMGB1 is secreted by immune cells and is known to be a cytokine mediator of inflammation. The CORO1A gene encodes the protein, coronin, actin binding protein 1A and is believed to regulate F-actin dynamics involved in phagocytosis [[28](#_ENREF_28)]. Interestingly, the GO category “Phagosome maturation” became significantly activated in both the MAP and MAA conditions although MAA magnitude of activation was larger. The CSK gene encodes the enzyme tyrosine-protein kinase. It is believed to be a negative regulator of many processes and that it is a key enzyme in the adaptive immune response.

The Phagosome pathway DBGGA analysis found this pathway to be strongly repressed during the early stage for MAA and then became strongly activated in the late stage for both MAA and MAP infected hosts. Employing our model interrogation method for Phagosome pathway, the most striking differences were found during the early stage for the gene relationship TUBA1A→TUBB and for the individual mechanistic genes FCGR1A and ITGA2. TUBA1A (tubulin alpha-1A chain) encodes a protein that is an essential component of the cytoskeletal microtubules. FCGR1A (high affinity immunoglobulin gamma Fc receptor I) is associated with phagocytosis which includes the membrane and cytoskeletal processes. These genes were significantly down regulated in MAA, but strongly up regulated in MAP infected host. In the innate immune system with mycobacteria, phagocytosis is reportedly facilitated by the mannose receptor (MRC) and complement receptors (CR) [[27](#_ENREF_27)]. The MAP infected host did not have any significant expression of MRC1 or CR1/2/3/4. However, the MAA infected host did have significant up regulated MRC1 and CR2.

**Th1 versus Th2 immune response**

Interestingly, it has been reported in some studies that mycobacterial evasion may involve the increase secretion of IL10 rather than IL12 by dendritic cells. Secretion of IL10 will favor the activation of a Th2 response which is incapable of destroying intracellular pathogens. The MAP infected host did, in fact, show IL10 expression and the strong activation of the GO category “Regulation of type 2 immune response” and the strong repression of the category “Regulation of T-helper 1 type immune response”, whereas, MAA favored IL12B expression combined with the activation of the “Regulation of T-helper 1 type immune response and the repression of “Type 2 immune response”. Another speculated evasion method involved the stimulation of antigen presenting cells with mycobacterial lipoprotein that resulted in the decrease in major histocompatibility class II complex (MHCII) expression [[29](#_ENREF_29)]. MHCII molecules are critical for the initiation of the antigen-specific immune response. In the MAP infected host, the MHCII gene BOLA-DMA was significantly down regulated while in the MAA infected host, the MHCII gene HLA-DOA was significantly up regulated.

**Difference in ABC transporter pathway**

The ABC transporter pathway has been associated with host immune defense mechanisms. The ABC transporter host pathway in the MAP condition was significantly perturbed in the early and late stages whereas in the MAA condition, the ABC transporter pathway was not perturbed at any time point. The early stage perturbation of the ABC transporter pathway for MAP condition was dominated by several genes of the ATP-binding cassette family. Specifically, the perturbation was induced by the up-regulated genes ABCA1 (ATP-binding cassette, sub-family A (ABC1), member 1), ABCC5 (ATP-binding cassette, sub-family C (CFTR/MRP), member 5), ABCC6 (ATP-binding cassette, sub-family C (CFTR/MRP), member 6), and ABCD3 (ATP-binding cassette, sub-family D (ALD), member 3) and the down-regulated genes ABCC3 (ATP-binding cassette, sub-family C (CFTR/MRP), member 3), ABCB8 (ATP-binding cassette, sub-family B (MDR/TAP), member 8), ABCB10 (ATP-binding cassette, sub-family B (MDR/TAP), member 10), ABCB4 (ATP-binding cassette, sub-family B (MDR/TAP), member 4) and ABCA3 (ATP-binding cassette, sub-family A (ABC1), member 3). In the late stage where adaptive immunity should be active, the ABC transporter perturbation was dominated by the strongly down-regulated genes ABCD3 which reversed direction from the early stage, and ABCB7 which was not significantly expressed in the early stage.
